# Optical and Photochemical Properties of Suwannee River Natural Organic Matter Fractions Generated by Preparative Free Flow Electrophoresis

**DOI:** 10.1021/acs.est.6c07200

**Published:** 2026-09-25

**Authors:** Garrett McKay, Kai Cheng, András Gáspár, Mourad Harir, Philippe Schmitt-Kopplin, Norbert Hertkorn, Ronald Benner, Karl Kaiser

**Affiliations:** † Zachry Department of Civil & Environmental Engineering, 14736 Texas A&M University, College Station, Texas 77843, United States; ‡ 9150 Research Unit Analytical Biogeochemistry, Helmholtz Munich, Neuherberg 85764, Germany; § Technical University of Munich, Chair of Analytical Food Chemistry, Freising 85354, Germany; ∥ Department of Thematic Studies−Environmental Change, Linköping University, Linköping SE 581 83, Sweden; ⊥ Department of Biological Sciences, 2629 University of South Carolina, Columbia, South Carolina 29208, United States; # Department of Marine and Coastal Environmental Sciences, 33604 Texas A&M University, Galveston, Texas 77554, United States; ∇ Department of Oceanography, Texas A&M University, College Station, Texas 77843, United States

**Keywords:** dissolved organic matter, electrophoresis, reactive oxygen species, structure reactivity relationships, fluorescence

## Abstract

Fractionation of dissolved organic matter (DOM) has been used for decades to simplify the challenging task of bulk sample characterization and to gain insight into the structural features governing DOM reactivity. Here, we used preparative free-flow electrophoresis (PFFE), which separates molecules based on their charge-to-size ratio, to separate Suwannee River natural organic matter (SRNOM) into fractions with varying electrophoretic mobility. Optical and photochemical data of the fractions were paired with extensive analytical characterization. The least electrophoretically mobile fraction had the lowest number-average molecular weight (*M*_n_), aromaticity, and specific ultraviolet absorbance (SUVA_254_), while showing the largest quantum yields of fluorescence as well as singlet oxygen and hydroxyl radical formation. Structure–activity relationships for the fractions, which exhibited substantial differences in mobility, aromaticity, and *M*_n_ did not follow the conventional trends observed in size-based DOM fractionation studies (e.g., SUVA_254_ ∝ aromaticity). These findings highlight the role of charge and structural differences in driving optical and photochemical properties apart from bulk aromaticity and *M*_n_. The distribution of absorbance, the sum of vanillyl and syringyl phenols, and DIOHBA (3,5-dihydroxybenzoic acid) indicates that the most electrophoretically mobile fractions with the highest absorbance are dominated by DIOHBA, a phenol monomer derived from tannins and flavonoids, rather than lignin. DIOHBA was inversely related to the fluorescence index and β/α. Overall, these results provide insight into SRNOM photophysics and highlight the utility of PFFE as a tool for resolving DOM structure–reactivity relationships.

## Graphical abstract

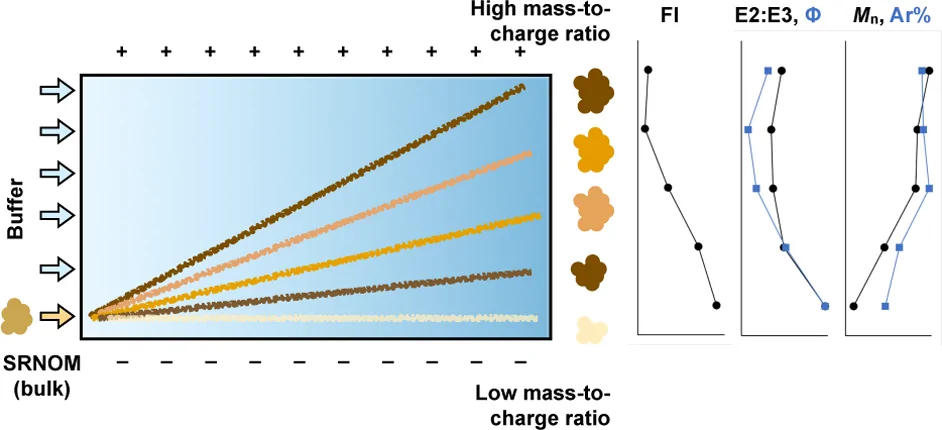

## Introduction

Dissolved organic matter (DOM) governs the mechanisms and rates of key processes in aquatic systems, such as elemental cycles,^1–5^ attenuation of contaminants,^6–8^ and productivity of the Earth’s oceans.^9,10^ Many of these processes are initiated by the absorption of sunlight by chromophoric DOM, which comprises up to ∼50% of the total DOM pool in terrestrial environments.^11^ DOM is an exceedingly complex mixture of molecules, rich in oxygenated functional groups, and sp^2^ hybridized carbon with varying molecular size.^12–15^ DOM molecular size and composition are altered during environmental processes, such as adsorption,^16^ oxidation,^17–19^ and coagulation.^20^

DOM structure impacts its reactivity in natural and engineered systems. One approach to resolving these structure–reactivity relationships is to fractionate DOM by size or polarity.^21^ It has been extensively demonstrated that molecular size is an important determinant of DOM optical properties^22,23^ and photoreactivity^13,24^ using size-based methods such as gel permeation chromatography,^12,25^ field-flow fractionation,^26,27^ and ultrafiltration.^13,24,28^ Although molecular weight depends only on the molecular structure, molecular size is a complex function of structure and environmental conditions such as pH and ionic strength.^29,30^ For example, ultrafiltration separates molecules based on size (weight is calibrated with globular proteins), but the resulting fractions are nonetheless often described as high- and low-molecular-weight fractions. It has been shown that lower molecular weight DOM generated from size-based separations has lower specific absorbance,^22,31^ larger spectral slope,^32^ higher fluorescence quantum yields (Φ_*f*_), blue-shifted emission, and higher quantum yields for the photoproduction of reactive intermediates.^13,15,24^ These lower molecular weight fractions also have a lower proportion of sp^2^-hybridized carbon than higher molecular weight fractions. Evidence for this comes both from nuclear magnetic resonance (NMR), which demonstrates a higher abundance of sp^2^ carbon, and Fourier transform ion cyclotron resonance mass spectrometry, which shows lower H/C ratios, higher double bond equivalence, and more formulas with an aromaticity index >0.5 for high relative to low molecular weight fractions.^13,31^ Several recent efforts have also focused on resolving DOM optical spectra via polarity-based separations with commercial solid-phase extraction cartridges.^33–35^

In contrast to separation by molecular size and polarity, DOM separation by charge density is less common. Capillary electrophoresis with optical or mass spectrometric detection demonstrated that electropherograms could act as a DOM fingerprint because the electrophoretic mobility is directly related to the charge and hydrodynamic friction of molecules in complex organic mixtures.^36^ In addition, Trubetskoj and coworkers investigated the optical and photochemical properties of DOM fractions of different electrophoretic mobility, which were generated by size exclusion chromatography (SEC) in the presence of 7 M urea.^25,37,38^ The predominant acidic functional groups in DOM are carboxylic acids and phenols,^39^ and the protonation state of these functional groups has been shown to alter DOM optical properties and photoreactivity.^40,41^ Although there are some reports employing absorbance and fluorescence detectors during capillary electrophoresis,^36^ complete spectral and photochemical characterization of DOM separated by charge-based methods is largely absent in the literature.

Here, we report the optical and photochemical properties of Suwannee River natural organic matter (SRNOM) fractions generated by preparative free-flow electrophoresis (PFFE) in a previous study.^42^ We characterized PFFE fractions and bulk SRNOM by optical measurements (absorbance and fluorescence), spectral pH titrations, fluorescence quenching, and measurements of apparent quantum yields for the photochemical production of singlet oxygen (^1^O_2_, Φ_Δ_) and hydroxyl radical (^•^OH, Φ_OH_). Correlations were examined between photochemical parameters and a wealth of compositional data, including NMR-derived functional group composition, molecular weight, amino acid content, and phenol monomers produced after copper oxide oxidation. A primary goal of this study is to assess the utility of PFFE as a DOM fractionation technique with respect to the molecular distinctness of its fractions. In addition, we expect that these data will provide new insight into the structural and compositional underpinnings of SRNOM optical and photochemical properties.

## Materials and Methods

### Preparative Free-Flow Electrophoresis

PFFE of SRNOM (1R101N) was performed in Munich using an Octopus PZE device manufactured by Dr. Weber GmbH (Kirchheim, Germany), as described in earlier studies.^42–45^ The device has a vertical separation chamber with dimensions of 500 mm (length) × 100 mm (width) × 0.5 mm (thickness), resulting in a chamber volume of 25 mL. The separation was performed at a temperature of 15 °C and a separation voltage of 500 V. The separation medium consisted of 5 mM ammonium carbonate (pH 9.2), while the electrode buffer consisted of 500 mM ammonium carbonate buffer at the same pH. The sample (20 mg mL^–1^ dry weight) was continuously pumped (2 mL h^–1^) into the bottom of the chamber and carried by the separation medium (965 mL h^–1^) in a laminar flow profile. At the top of the chamber, the separation medium and sample were collected in a tray containing 96 wells.

Solutions from wells were binned into 11 fractions, and fractions were lyophilized to obtain solid for further analysis and to remove ammonium carbonate. The appearance of a dry, fluffy powder after lyophilization is strong evidence of the removal of ammonium carbonate. Although 11 fractions were collected during PFFE, only five were analyzed in detail here, as they are the main contributors to the absorbance detected in the electrophoretogram.

### Analytical Methods

The SRNOM fractions and their analytical characterization were originally described by Gáspár,^42^ which included NMR and amino acid analyses. A brief description of all analytical methods is provided here (see complete details in the Supporting Information). Optical measurements, SEC, and photochemical characterization were performed in 2020 and are original to this work.

Optical measurements, SEC, and photochemical characterization were completed at Texas A&M. Briefly, absorbance and fluorescence spectra were collected with an Aqualog (Horiba). Standard protocols for data treatment were implemented, such as blank subtraction, inner-filter corrections, Raman normalization, and Rayleigh masking.^46,47^ Optical surrogates were calculated according to Korak and McKay^48^ and included the specific ultraviolet absorbance at 254 nm (SUVA_254_), E2:E3 ratio, spectral slope between 275 and 290 (*S*_275–290_) and 350–400 (*S*_350–400_), the fluorescence index (FI), and the freshness index (β/α). SEC was completed with an Ultimate 3000 UHPLC (Thermo Fisher) and Waters Protein-Pak (15 × 300 mm) column, with a mobile phase consisting of 0.1 M potassium chloride, 2 mM potassium dihydrogen phosphate, and 2 mM potassium hydrogen phosphate at pH 6.8. The flow rate was 1.0 mL min^–1^. Retention time was calibrated to molecular weight using polystyrenesulfonates (PSS) ranging from 246 to 18 000 Da. SEC is one of the most common techniques used in the DOM community for obtaining apparent molecular weights.^12,49,50^ Although PSS do not perfectly represent DOM size, number-average molecular weights (*M*_n_) measured by SEC with PSS calibration agree well with values from vapor pressure osmometry.^23,51^ Total organic carbon was measured with a Shimadzu V-SCH organic carbon analyzer on 0.45 μm-filtered samples using the non-purgeable organic carbon method.

NMR analysis was performed in Munich, Germany, using a Bruker DMX spectrometer (*B*_0_ = 11.7 T). We focus here on ^13^C data, which were collected for PFFE fractions (5–35 mg) redissolved in up to 135 μL CD_3_OD (99.96% ^3^H, Merck, Darmstadt, Germany) at 283 K. Shift regions were integrated to obtain quantitative estimates of functional group distributions (see Text S1).

Amino acid analysis was performed in Columbia, South Carolina, while copper oxide oxidation and phenol monomer analysis were performed in Galveston, TX. Briefly, amino acid content was measured on hydrolyzed samples (6 M HCl, 20 h, 110 °C) by HPLC after in-line derivatization with *o*-phthaldialdehyde/*N*-isobutyryl-l-cysteine or *o*-phthaldialdehyde/*N*-isobutyryl-d-cysteine.^52^ Quantification of phenol monomers after copper oxide oxidation was performed by GC/MS following cleanup with solid-phase extraction (Oasis HLB cartridges). *p*-Hydroxyphenyl (P), vanillyl (V), syringyl (S), and cinnamyl (C) phenols were quantified, including *p*-hydroxybenzaldehyde (PAL), *p*-hydroxyacetophenone (PON), *p*-hydroxybenzoic acid (PAD), vanillin (VAL), acetovanillone (VON), vanillic acid (VAD), syringaldehyde (SAL), acetosyringone (SON), syringic acid (SAD), *p*-coumaric acid (CAD), and ferulic acid (FAD). The sum of V and S phenols (Σ_6_ = VAL + VON + VAD + SAL + SON + SAD) is a strong indicator of lignin content. 3,5-Dihydroxybenzoic acid (DIOHBA) was also measured.

### Solution Preparation

Solutions for SEC, optical, and photochemical analyses were prepared using ultrapure water (>18.0 MΩ cm) from a Barnstead water system (Thermo Fisher). PFFE fractions and bulk SRNOM were dissolved in ultrapure water. Sample pH was adjusted to 7.0 with 0.1 M NaOH, and solutions were stirred overnight and filtered with pre-rinsed 0.45 μm polyether sulfone syringe filters. These stock solutions were stored at 4 °C until dilution for subsequent experiments. Dilutions were prepared in either 5 mM potassium phosphate buffer at pH 7.2 (for optical and photochemical measurements) or, for fluorescence quenching (see Text S1), in acetic acid (pH 4) or sodium carbonate (pH 9).

#### Photochemical Measurements

A more detailed description of the probe compound methods is provided in Text S2. A Rayonet merry-go-round photoreactor with 12 365 nm bulbs, equipped with a fan, was employed for all irradiations, which were conducted in borosilicate glass tubes (effective path length of 0.84 cm). Briefly, 25 μM furfuryl alcohol and 2 mM benzoate were used for quantifying the formation rates of ^1^O_2_ and ^•^OH, respectively. The rate of ^•^OH formation was derived from the rate of salicylate formation using the yield of 0.204 mol salicylate per mol ^•^OH.^53^ Apparent quantum yields were calculated using the measured ^1^O_2_ or ^•^OH formation rate and the rate of light absorption, the latter calculated using the photon irradiance (measured with *p*-nitroanisole/pyridine^54^), solution absorbance, and path length in the quartz tube. Formation of DOM triplet states was not measured in this study (e.g., using trimethylphenol) as ^1^O_2_ is considered an indicator of triplet reactivity.^55^

Although commonly used within the aquatic photochemistry literature, the 365 nm light source in our study likely results in higher Φ_Δ_ values compared to a solar simulator due to Φ_Δ_’s wavelength dependence.^56^ However, our objective was to investigate fundamental structural features governing SRNOM optical properties and photochemistry, not to accurately represent environmental polychromatic conditions.

#### Statistical Methods

Correlation analysis was performed using the Statistics and Machine Learning Toolbox in MATLAB 2024b. Pearson correlation coefficients and their associated *P* values were calculated. Correlations with *a P*-value of 0.01 (indicated with an *) were classified as significant due to the small number of points in each correlation (*n* = 6). Correlations with 0.01 ≤ *P* < 0.05 are considered suggestive trends, and the *ρ_P_* values are still shown.

## Results

### Preparative Free-Flow Electrophoresis Produces SRNOM Fractions with Varying Optical and Photochemical Properties

PFFE separates molecules based on size and charge, with a molecule’s effective electrophoretic mobility being proportional to its charge-to-size ratio. Higher charge density translates into a higher velocity of the ions in solution in the electric field and thus effective electrophoretic mobility (field-strength-normalized ion velocity).^57^ The electropherogram demonstrates that the majority of SRNOM molecules have a narrow range of electrophoretic mobilities, between −0.03 and −0.005 cm^2^ *V*_min_^–1^ (Figure 1). Although only five fractions are discussed below (Fr6–Fr10), the mass of organic carbon (OC) in all 11 fractions (Fr1–Fr11) was measured to assess the mass balance (Table S2). Approximately 80% of the total SRNOM mass was recovered, while slightly less of the OC was recovered (73%), which could be explained by the incomplete removal of ammonium carbonate. Relative to the mass quantified in the 11 fractions, fractions 6 through 10 comprise more than 95% of the SRNOM and OC mass as well as most of the absorbance at 254 nm in the electrophoretogram (Figures 1 and S1). The unrecovered mass may include high-mobility molecules such as small, aliphatic carboxylic acids. Although we did not examine the organic carbon concentration in each tube after PFFE, an early report by Leenheer and Malcolm^57^ shows a substantial organic carbon peak that is weakly UV-absorbing with high electrophoretic mobility. Given the probable chemical nature of the missing mass, we do not expect that its absence will impact the optical and photochemical properties observed here.

**Figure 1:**
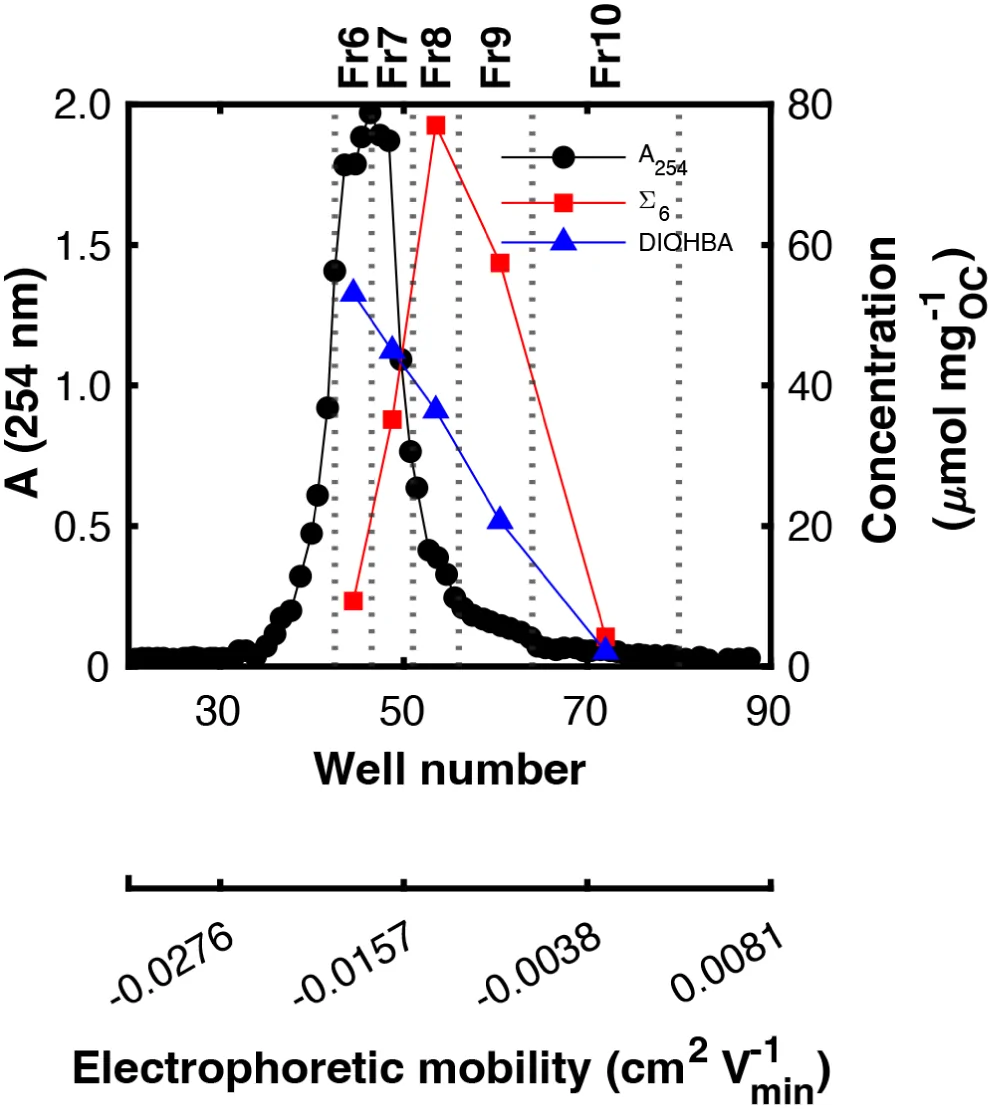
Electropherogram of SRNOM showing absorbance at 254 nm (left axis) and the sum of vanillyl and syringyl lignin phenols (Σ_6_) and 3,5-dihydroxybenzoic acid (DIOHBA) (right axis).

PFFE of SRNOM produces fractions with distinct chemical, optical, and photochemical properties (Figures 1 and 2). The least mobile Fr10 has the lowest SUVA_254_ (0.98 L mg_C_^–1^ m^–1^), highest FI (1.58) and *β*/α (0.831), lowest *M*_n_ (344 Da), and lowest abundance of aromatic plus phenolic carbon (herein referred to as aromaticity) (24.8%). Although there is a general trend of decreasing *M*_n_ and aromaticity with decreasing mobility (Fr6 → Fr10), the first three fractions are largely invariant in these properties. Absorbance-based surrogates (SUVA_254_ and E2:E3) and Φ_*f*_ do not show a consistent trend with decreasing mobility (Figure 2B and D). In contrast, FI and *β*/α (fluorescence-based surrogates) generally increase with decreasing mobility (Figure 2C). Fr10 has the highest Φ_*f*_, Φ_Δ_, and Φ_OH_, Fr7 has the lowest values, and values for Fr6 and Fr8 are slightly larger than those for Fr7 (Figure 2D–F). This pattern matches the trend of E2:E3.

**Figure 2:**
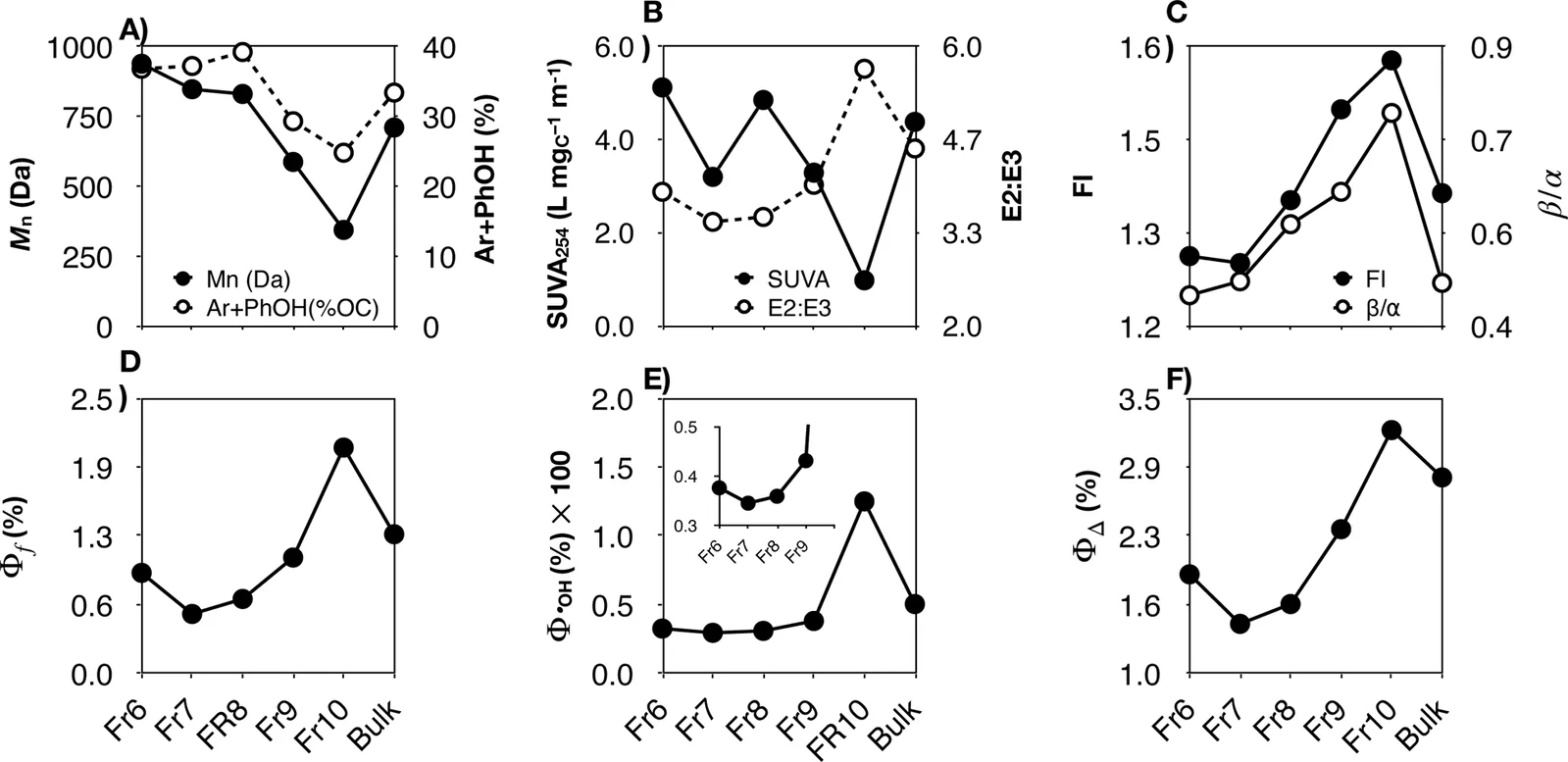
Physicochemical properties, optical surrogates, and apparent quantum yields of PFFE fractions and bulk SRNOM. (A) Number-average molecular weight (*M*_n_) and the sum of aromatic and phenolic resonances (108–167 ppm) from ^13^C-NMR. (B) Specific ultraviolet absorbance at 254 nm (SUVA_254_) and the E2:E3 ratio (*A*_250_/*A*_365_). (C) Fluorescence index (FI), quantified as the ratio of emission intensity at 470 to 520 nm at 370 nm excitation, and *β*/*α*, quantified as the ratio of emission at 380 to 420–435 nm (max is selected) at 310 nm excitation. Additional details are provided in Korak and McKay.^48,58^ Apparent quantum yields of (D) fluorescence (Φ_*f*_) at 370 nm excitation, (E) hydroxyl radical (Φ*_OH)_*, and (F) singlet oxygen (Φ_Δ_). Details on apparent quantum yield determinations are provided in Text S2.

These results contrast with prior reports employing ultrafiltration, which typically show continual changes in optical surrogates and photochemistry with varying nominal molecular-weight cutoff of the ultrafiltration membrane.^13,15,24^ For example, Fr7 has the second highest *M*_n_ and aromaticity but the second lowest SUVA_254_ (3.2 L mg C^–1^ m^–1^), while Fr6 has the highest SUVA_254_ and highest *M*_n_ (938 Da). Fr7 and Fr9 have different aromaticity and *M*_n_ but similar SUVA_254_ (Figure 2A and B). Although chemical composition and molecular weight are not directly measured in all ultrafiltration studies, most of those that measure these parameters (e.g., by mass spectrometry for aromaticity or SEC for molecular weight) report that permeate DOM has lower aromaticity and molecular weight.^13^

A conservative mixing model was applied to the data in which the signal for bulk SRNOM $\left(S_{\mathrm{bulk}}^{\mathrm{calc}}\right)$ was calculated based on the fractional mass (*f_i_*) and each fraction’s signal (*S*_*i*_) such that $S_{\mathrm{bulk}}^{\mathrm{calc}}=\Sigma_{i=1}^{5}f_{i}S_{i}$ (Figure 3). Additional details are shown in Text S3. When *f_i_* is based on total mass, $S_{\mathrm{bulk}}^{\mathrm{calc}}$ underestimated measured signals by between 14 and 53% $\left(\left[S_{\mathrm{bulk}}^{\mathrm{calc}}-S_{\mathrm{bulk}}^{\mathrm{meas}}\right]/S_{\mathrm{bulk}}^{\mathrm{meas}}\times 100\right)$ . In contrast, model calculations are closer to measured signals when the total mass of OC is used to calculate *f_i_*, with percent differences between −41 and +5%. When using OC mass for *f_i_*, differences between modeled and calculated FI, *M*_n_, aromaticity, and λ_pH4_ are greater than −10% while SUVA_254_ has a −13% difference. Φ_*f*_, Φ_Δ_, and Φ_OH_ exhibit the poorest agreement between model and measured signals, with model values underestimating the measured signals by 30% or more.

**Figure 3:**
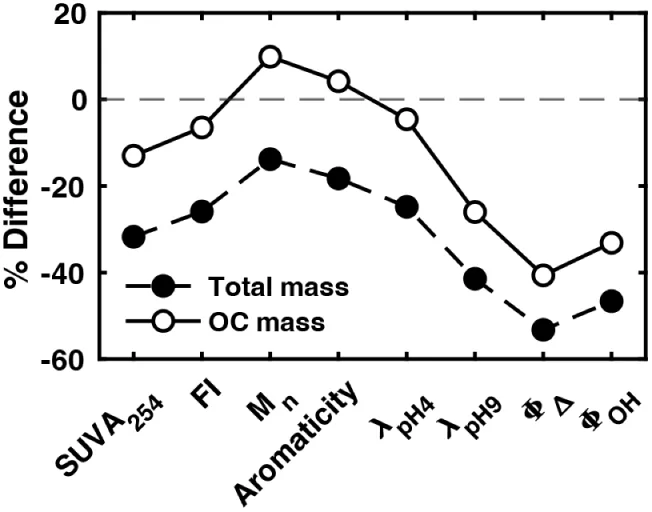
Differences between conservative mixing model calculations and measured values $(\%\;\mathrm{difference}=\left[S_{\mathrm{bulk}}^{\mathrm{calc}}-S_{\mathrm{bulk}}^{\mathrm{meas}}]/S_{\mathrm{bulk}}^{\mathrm{meas}}\times 100\right)$ for selected optical surrogates, physicochemical data, and quantum yields.

The disagreement between the conservative mixing model and bulk sample measurements suggests nonadditive interactions in bulk SRNOM that are not retained in PFFE fractions. The incomplete mass balance obtained during PFFE should be noted as a caveat warranting future examination. Nevertheless, bulk values are within the range of PFFE fractions, indicating that fractionation captures the full extent of compositional variability.

### Fluorescence Quenching and Spectral pH Titration Reveal Charge-Density Differences of Chromophores in PFFE Fractions

PFFE does not discriminate between chromophoric and nonchromophoric molecules. Thus, one would expect that the chromophores present in Fr6 would have greater charge-to-size ratios than those of later fractions. Fr6 also has the largest measured molecular weight (Figure 2A); thus, it should have a far greater number of charged sites than other fractions to have the largest charge density and electrophoretic mobility.

pH titrations and fluorescence quenching were used to evaluate the charge density of chromophores in the PFFE fractions. Fluorescence quenching with tempamine (TA) and tempol (TO) provides an assessment of the surface charge of fluorophores.^59,60^ The ratio of Stern–Volmer constants for each quencher (*K*_TA_/*K*_TO_), defined as λ, is related to the apparent attractive electrostatic potential (Ψ′) via λ = exp­(−Ψ′/*k*_B_ *T*), where *k*_B_ is the Boltzmann constant and *T* is the temperature. Another way of conceptualizing λ is as the ratio of local to bulk quencher, which is unity for neutral tempol and >1 for cationic tempamine. The measured value of λ is lower than the actual value due to dynamic quenching processes. Nonetheless, λ is a useful measure for assessing Ψ′^′^.^59^

λ values for all PFFE fractions are smaller at pH 4 than at pH 9 (Figure 4), consistent with the larger expected charge density at pH 9 due to a greater fraction of deprotonated carboxylic acids and phenols.^39^ λ at pH 4 and 9 decrease with decreasing electrophoretic mobility, consistent with Fr6 having the highest charge-to-size ratio and therefore the largest attractive electrostatic potential. The difference in λ between pH 4 and 9 for each fraction is largest for Fr6 and bulk SRNOM, whereas λ are more similar at each pH for Fr8 through Fr10. Overall, the quenching behavior of SRNOM fractions corroborates the effective separation of fluorophores by PFFE into molecules with varying charge density.

**Figure 4:**
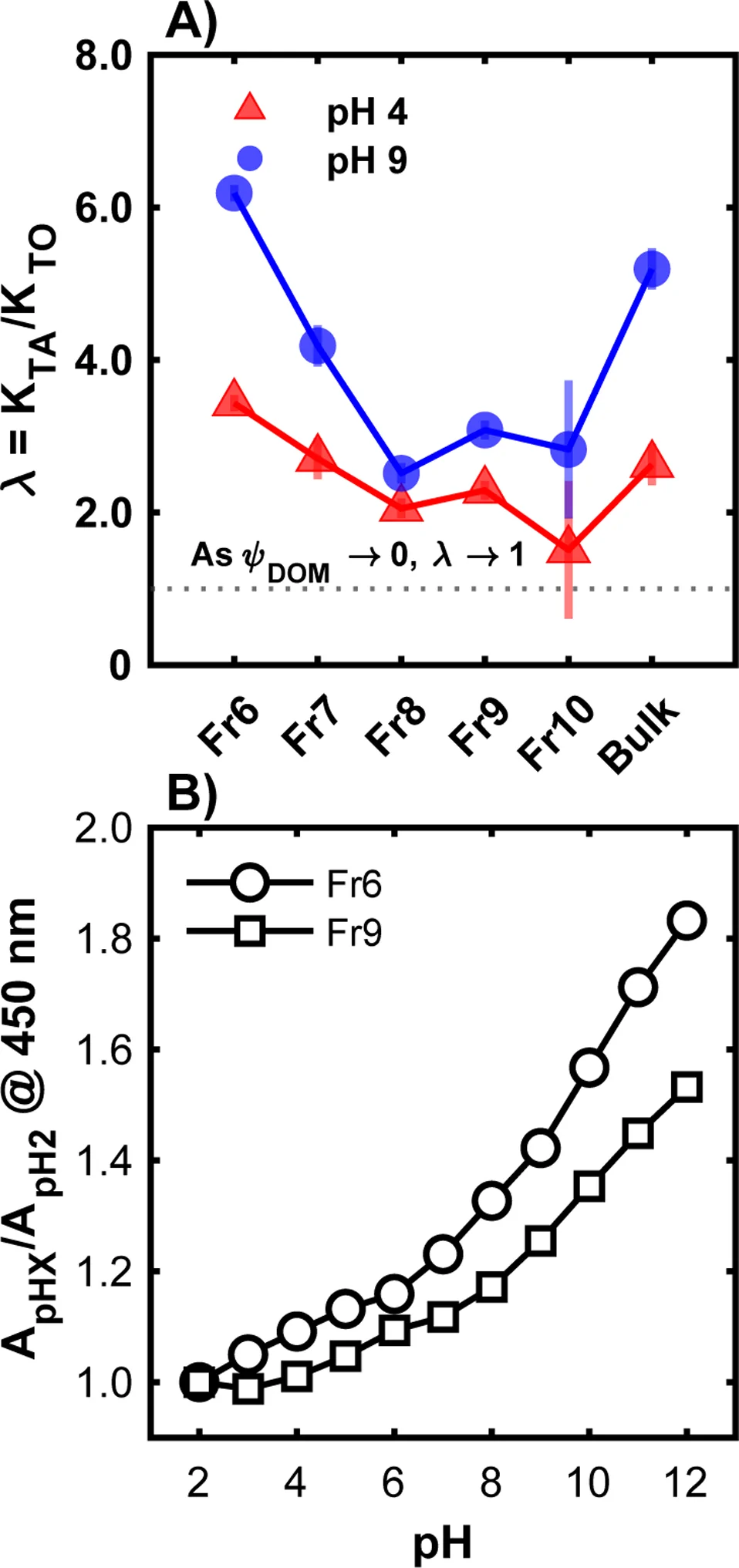
Characterization of charged chromophores and fluorophores in PFFE fractions. (A) Ratio of Stern–Volmer constants for quenching by cationic tempamine (*K*_TA_) and neutral tempol (*K*_TO_) at pH 4 (buffered by acetic acid) and pH 9 (buffered by carbonate). The term *λ* is related to the apparent attractive electrostatic potential of the fluorophore being quenched by *λ* = exp­(−ψ/*k*_B_ *T*). Symbols represent the ratio of Stern–Volmer constants derived from model fitting, and error bars show propagated error resulting from uncertainties in the individual constants. Stern–Volmer fitting details are provided in Text S1. (B) Absorbance at 450 nm relative to pH 2 for Fr6 and Fr9. Samples were adjusted to pH 2 with 3 M HCl and titrated to pH 12 with small additions of 1 M NaOH. pH titrations were not performed for Fr7, Fr8, and Fr10 due to limited sample quantities.

Spectral pH titrations provide an additional assessment of the chromophore charge density. The response of absorbance to an increase in pH has been linked to the roles of carboxyl- and phenol-containing chromophores.^40,41,61^ The ratio of absorbance at 450 nm as a function of pH normalized to pH 2 (*A*_pHX_/*A*_pH2_) increases more rapidly with increasing pH for Fr6 relative to Fr9 across the pH 2 to 6 (carboxyl) and pH 8 to 12 (phenol) ranges (Figure 3B). Thus, the pH-dependent absorbance behavior of PFFE fractions further corroborates the effective separation of chromophores within SRNOM.

### Relationships between Optical Surrogates and Compositions

Relationships between compositional data and photochemical properties were assessed for bulk SRNOM and PFFE fractions using Pearson correlation coefficients (*ρ_P_*). The compositional data included NMR-derived functional group composition, SEC estimates of molecular weight, and the abundance of phenol monomers released during copper oxide oxidation. Amino acid yield is also included as it is often related to fluorescent DOM to infer information about source and level of processing.^62 13^C-NMR-derived functional group assignments, phenol monomer content, and amino acid yield are normalized to the mass of organic carbon in each fraction. Pearson was chosen over Spearman rank coefficients because the most interesting and relevant scatter plots exhibited linearity. The choice of Pearson vs. Spearman does result in differences in the correlation coefficient, but *ρ_P_* and *ρ_S_* exhibit a roughly one-to-one relationship (Figure S2). Differences in *P*-values between the two analyses can impact interpretations. For example, the FI-aromaticity relationship exhibits good linearity (*R* ^2^ = 0.816) and a significant Pearson correlation coefficient (*ρ*_P_ = −0.91 and *P* < 0.01) but the calculated Spearman rank coefficient (ρ*_S_* = −0.83) has *P* > 0.01.

Figure 5A shows a heatmap of correlation coefficients, while Figure 5B–U displays selected scatter plots. *ρ_P_* with *P*-values <0.05 are printed, *ρ_P_* with *P*-values <0.01 are marked with an ∗, and those with *P* > 0.05 are not displayed. Considering structural characteristics from each category, aromatic and carbonyl carbon, amino acid yield, *M*_n_, and DIOHBA exhibited the highest frequency of correlations with optical surrogates with *P* < 0.05. FI had eight correlations with *P* < 0.05 and five with *P* < 0.01, which was the most of any of the optical surrogates or quantum yields.

**Figure 5:**
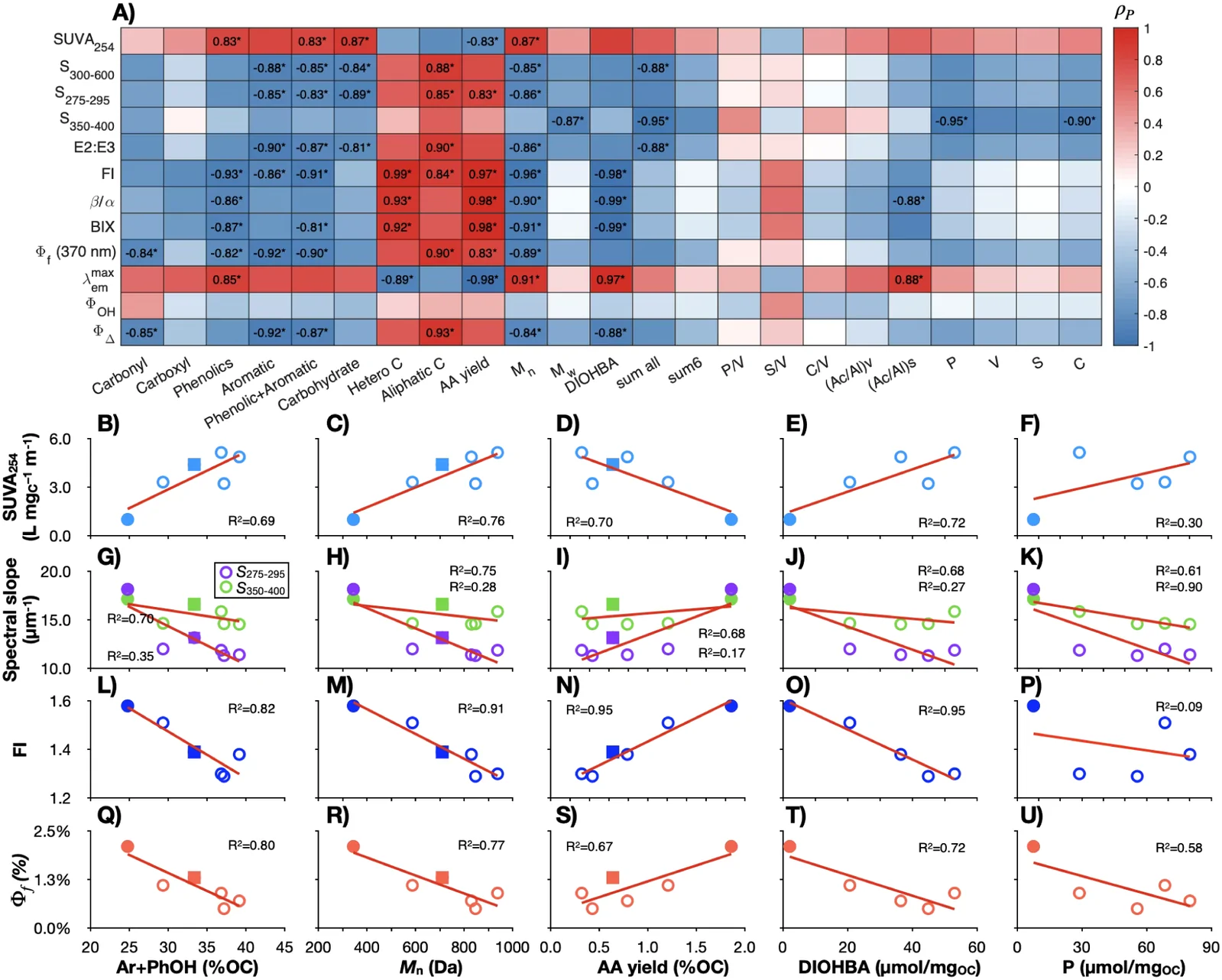
Relationships between optical surrogates and physicochemical properties of SRNOM fractions. (A) Heatmap represents the value of the Pearson rank correlation coefficient (*ρ*_P_). *ρ_P_* values are printed in the heatmap if *P* < 0.05 and are marked with an * if *P* < 0.01. (B–U) Closed circles represent Fr10, closed squares represent bulk SRNOM, and open symbols represent Fr6–Fr9. Ar + PhOH (% OC) is the percentage of organic carbon in the ^13^C-NMR spectrum with resonances between 108 and 167 ppm, *M*_n_ is the SEC-derived number-average molecular weight, AA yield (% OC) is the percentage of the organic carbon mass attributable to quantified amino acids, DIOHBA is the carbon-normalized amount of 3,5-dihydroxybenzoic acid, and *P* is the sum of the carbon-normalized amounts of *p*-hydroxybenzaldehyde, *p*-hydroxyacetophenone, and *p*-hydroxybenzoic acid.

Examining the correlation coefficients in Figure 5A might suggest that the relationships between composition and optical or photochemical properties are consistent with original studies linking DOM composition to optical surrogates. For example, SUVA_254_ is positively correlated to aromaticity and negatively correlated to *M*_n_ (Figure 5B and C), consistent with Weishaar et al.^63^ and Chin et al.^12^ (for diverse hydrophobic organic acid isolates) and ultrafiltration studies;^13,24^ spectral slope (*S*_275–295_) and E2:E3 are negatively correlated to aromaticity and *M*_n_, consistent with past work on fractionated DOM (Figure 5A, G, and H);^22,31^ and Φ_Δ_ is negatively correlated to *M*_n_, consistent with ultrafiltration studies.^13,24^

Despite the appearance of significance based on *ρ_P_* values, scrutiny of individual scatter plots is necessary to decipher which relationships are meaningful. Selected scatter plots with a linear regression fit and *R* ^2^ values are shown in Figure 5B–U. By far, the tightest linear relationships are for FI, with *R* ^2^ > 0.9 for *M*_n_, amino acid yield, and DIOHBA content. DIOHBA shows the highest frequency of correlations with *P* < 0.05 (four have *P* < 0.01) among the phenol monomers, being negatively correlated to *β*/*α* (Figure S3) and Φ_Δ_ (Figure 5T). *S*_275–295_ is not significantly correlated to any phenol monomer (*P* > 0.01) but is significantly related to aromaticity (*P* = 0.0388) and *M*_n_ (*P* = 0.0269) (Figure 5G and H). This contrasts with the results of Fichot and co-workers, which show a strong exponential relationship between *S*_275–295_ and the sum of *p*-hydroxy, vanillyl, and syringyl phenols in whole water samples from the northern Gulf of Mexico^64^ and coastal areas worldwide.^65^ This is most likely due to the more limited range of *S*_275–295_ values in our study (10–18 μm^–1^) compared to Fichot (10–40 μm^–1^) which included photobleached samples with higher *S*_275–295_. *S*_350–400_ is well correlated to *p*-hydroxyphenyl (*P* = 0.0132) and cinnamyl (*P* = 0.0361) phenols (*ρ_P_* < −0.9) but not aromaticity or *M*_n_ (*P* > 0.2). None of the relationships between phenol monomers and E2:E3 or SUVA_254_ are significant, although we should note that the *P*-value for the SUVA_254_-DIOHBA correlation is 0.070. DIOHBA has been interpreted as an indicator of tannins and flavonoids,^66^ distinct from the syringyl and vanillyl phenols traditionally considered surrogates for lignin.

Interestingly, amino acid yield shows a strong relationship to FI and *β*/*α* (*P* ≤ 0.001 *ρ_P_* > 0.97), which are surrogates for DOM source and age.^62^ This finding complements the results of Yamashita et al.,^67^ who reported a positive correlation between a tryptophan-like parallel factor analysis component and amino acid yield in the northern Gulf of Mexico. Our results for PFFE fractions suggest that this correlation occurs for regions of the excitation–emission matrix that are less prone to low fluorescence intensities.

### Relationships between ^1^O_2_ and ^*•*^OH Quantum Yields and DOM Structure

Φ_Δ_ varies by over two-fold between the fractions (0.0145 to 0.0321), whereas Φ_OH_ values are largely similar except for Fr10 (1.2 × 10^–4^) which is four-fold larger than the lowest value, Fr7 (2.9 × 10^–5^). Φ_Δ_ is correlated to aromaticity (*P* < 0.01), carbonyl content (*P* = 0.0336), and *M*_n_ (*P* = 0.0363). In contrast, Φ_OH_ is not significantly correlated to any of the compositional parameters (*P*> 0.01).^68^ Scatter plots of these data with a linear regression model are shown in Figure S5. The negative dependence of Φ_Δ_ on both aromaticity and *M*_n_ is consistent with previous studies employing ultrafiltration,^13,24^ while the negative relationship to carbonyl content has, to our knowledge, not been reported previously.

### Discussion

#### PFFE Produces Fractions with Distinct Structures but Similar Optical and Photochemical Properties

A primary finding of this research is that the molecular structure, composition, and photoactivity of PFFE fractions appear to differ from size-based fractionation techniques such as ultrafiltration. Studies employing ultrafiltration typically show increasing E2:E3 with decreasing molecular weight, as defined by the nominal molecular weight cutoffs of the ultrafilters. For example, Maizel and Remucal demonstrate that ultrafiltration permeates of Pony Lake and Suwannee River fulvic acid have larger E2:E3 values than both the bulk sample and retentate, and show a concomitant increase in the abundance of formulas with an aromaticity index >0.5.^13^ Likewise, Mostafa et al. show that ultrafiltration permeates of SRNOM and a wastewater derived DOM have longer SEC retention times and larger E2:E3 values than their respective bulk and retentate fractions.^24^ In contrast, PFFE fractions of SRNOM show minimal change in E2:E3 from Fr6 through Fr9, which vary in molecular weight by nearly two-fold and aromaticity by 1.5-fold. There is no consistent trend in SUVA_254_ from Fr6–Fr9; however, Fr10 has the lowest *M*_n_, aromaticity, and highest E2:E3 of all fractions. In general, absorbance-based optical surrogates that characterize spectral slope do not vary considerably until Fr10. Surprisingly, Φ_*f*_ displays a pattern similar to E2:E3. This contrasts with FI and *β*/*α* which generally increase with decreasing electrophoretic mobility and exhibit tight correlations to aromaticity and *M*_n_.

Trubetskoj and coworkers investigated the optical properties and photoreactivity of DOM fractions with varying electrophoretic mobility,^25,37,69^ and some of their observations align with those from PFFE. In Trubetskoj’s studies, DOM was separated by low-pressure SEC in the presence of 7 M urea and the electrophoretic mobility of the fractions was measured post hoc with gel electrophoresis.^38^ They observed that the most electrophoretically mobile fractions (C + D) had higher SUVA_280_ than the least mobile fractions (A and B).^25,37^ They also showed that the more mobile fractions had similar E2:E3,^25^ similar to Fr6–Fr9 in our study. One discrepancy is that, for PFFE separation, fractions with higher electrophoretic mobility also have the largest molecular size, whereas Trubetskoj et al. found that the most mobile fraction had the smallest molecular size.^25^ In addition, Trubetskoj et al. observed that the most electrophoretically mobile fraction had higher photosensitizing efficiency,^37,69^ which is opposite to the result seen here that Fr10 has the largest Φ_Δ_ and Φ_OH_ values. Although speculative, these discrepancies could be due to disruption of intermolecular interactions (e.g., H-bonding) by 7 M urea in the most electrophoretically mobile fractions that have a high abundance of H-bond donors and acceptors.^69^ Such disruptions could decrease the apparent size of the DOM, as measured by SEC, leading to higher photosensitization efficiency of the high-mobility fraction.^24^ Trubetskoj et al. dialyzed samples prior to photochemical measurements; thus, any disaggregation resulting from 7 M urea would need to be preserved post-dialysis for this explanation to be valid. In addition, because Trubetskoj and coworkers characterized electrophoretic mobility after SEC, separation by charge density (i.e., pulling apart molecules with similar size but different charge) would not have occurred during SEC. Clearly, more work is needed to evaluate these hypotheses. Regardless, the agreement between our study and Trubetskoj et al. that high-mobility fractions may have similar absorbance-based properties further supports the differences between charge-based separation and ultrafiltration.

We propose two possible explanations for the lack of systematic relationships between aromaticity, *M*_n_, and absorbance-based surrogates among PFFE fractions. First, aromaticity and *M*_n_ are coarse descriptors that do not fully capture the nuances of DOM structure. Separation by charge-density results in high-mobility fractions having a greater abundance of ionizable functional groups (Table S3). Spectra of fractions with hhigher charge density will be impacted by these additional functional groups in ways not easily captured with bulk measures of aromaticity and molecular weight. For example, phenol and acetovanillone have the same number of aromatic carbon atoms but quite different absorbance and fluorescence spectra due to the electronic nature of their substituents.^41^ The more mobile fractions exhibit red-shifted spectra relative to lower-mobility fractions (Table S4, Figure S6). This observation is consistent with the positive correlation between fluorescence asymmetry (a measure of red-shifted emission) and the relative abundance of aromatic and highly oxygenated formulas reported by Nikolaev and coworkers.^33^ Nikolaev and coworkers further hypothesized that this relationship is driven by the proportion of oxidized aromatic compounds with low p*K*_a_ values, which are most likely to be carboxylic acids. These same structures govern separation of DOM molecules by PFFE and are more abundant in the high-mobility fractions (Table S3).

A second explanation is related to the PFFE separation mechanism. Ultrafiltration separates DOM primarily on the basis of size, whereas PFFE separates DOM on the basis of size and charge. Thus, molecules with the same molecular weight but different charge densities will be separated in PFFE but not by ultrafiltration. This explains why Fr6 through Fr8 can have varying charge densities but similar *M*_n_. The additional separation dimension helps to resolve compositional heterogeneity among molecules with overlapping molecular weights.

In contrast to absorbance-based surrogates, which do not vary systematically with electrophoretic mobility and exhibit weaker correlations to aromaticity and *M*_n_, FI and *β*/*α* show both a consistent increase with increasing electrophoretic mobility and have tight correlations to *M*_n_ and aromaticity. The strong inverse correlation of these fluorescence-based surrogates *to M*_n_ and aromaticity is consistent with the genesis studies of these optical surrogates as well as continued inquiry using ultrafiltered samples.^58,70–72^ It has been well-documented that FI and *β*/*α* are inversely related to the maximum emission wavelength $\left(\lambda_{\mathrm{em}}^{\max}\right)$ at the excitation wavelength used to determine these surrogates (see Figure S4).^48,73^ Because $\lambda_{\mathrm{em}}^{\max}$ is inversely proportional to the energy gap between the first excited and ground electronic states,^74^ this result suggests that fluorophores in more mobile fractions emit from lower energy states.

#### The Importance of DIOHBA

Another explanation for the lack of systematic relationship between structure and absorbance-based surrogates for SRNOM fractions is derived from the observation that Σ_6_ and DIOHBA have different distributions in the PFFE fractions (Figure 1). DIOHBA is most abundant (i.e., μmol mg_OC_^–1^) in Fr6 and decreases monotonically with decreasing mobility, whereas Σ_6_ increases from Fr6 to Fr8 and decreases thereafter. Importantly, the same mobility pattern holds when absolute DIOHBA and Σ_6_ amounts (i.e., μmol) are compared to the electropherogram (Figure S9). We hypothesize that differences in the distribution of Σ_6_ and DIOHBA impact the structure of the aromatic chromophores in each fraction. Lignin phenol concentrations, which do not include DIOHBA, are often considered the main predictor of chromophoric DOM in natural waters^64,75,76^ and are used as a tracer of terrigenous DOM.^64^ In contrast, our results suggest that material with the highest absorbance in the electropherogram (Fr6) is dominated by DIOHBA.

In addition, DIOHBA abundance has a strong inverse correlation to FI and *β*/*α*. This study is the first to our knowledge to report significant correlations between DOM optical surrogates and DIOHBA. In contrast, studies of terrestrial^77^ and coastal systems^64,65,67^ have reported both intrinsic^64,77^ and extrinsic^65,67,75^ correlations to lignin phenol composition. For the SRNOM fractions, Σ_6_ was correlated only to *S*_350–400_. Combined, these observations strongly indicate that DIOHBA precursors are a dominant force in shaping the fluorophores governing FI and *β*/*α*, while lignin phenols play a role in *S*_350–400_.

DIOHBA is often interpreted as derived from tannins and flavonoids as opposed to lignin.^66^ Alkali-extracted lignin has Φ_*f*_ values much lower and $\lambda_{\mathrm{em}}^{\max}$ values higher than DOM isolates.^78^ Tannic acid fluorescence is complicated by self-oxidation in water, and model compounds for tannin and lignin tend to have higher Φ_*f*_ than the bulk materials.^11,79^ Insight into compositional differences of DIOHBA-dominated Fr6 and lignin-dominated Fr9 comes from examining the intensity-weighted emission wavelength $\left(\lambda_{\mathrm{em}}^{\mathrm{avg}}\right)$ of these fractions and bulk SRNOM as a function of pH (Figure S10). The pH-dependent $\lambda_{\mathrm{em}}^{\mathrm{avg}}$ of bulk SRNOM more closely matches the behavior of Fr9 at λ*_ex_* shorter than ∼290 nm wherein a slight red shift is observed across the carboxyl pH range (∼2 to 6). In contrast, Fr6 $\lambda_{\mathrm{em}}^{\mathrm{avg}}$ values are comparatively constant across all pH at these excitation wavelengths. At λ_ex_ of 300 and 310 nm, there is little change in $\lambda_{\mathrm{em}}^{\mathrm{avg}}$ for bulk SRNOM and Fr6, while Fr9 emission red shifts from pH 2 to 6. At UVC excitation wavelengths, both bulk SRNOM and Fr6 emission spectra blue shift with increasing pH, while average emission for Fr9 remains relatively constant. Thus, the pH-dependent behavior of bulk SRNOM more closely resembles DIOHBA-dominant Fr6 at λ_ex_ longer than ∼300 nm and more closely resembles Σ_6_-dominant Fr9 at λ*_ex_* shorter than ∼300 nm.

These observations show how different structures in Fr6 and Fr9 contribute to SRNOM fluorescence behavior at different excitation wavelengths. Given the strong relationships observed here between PFFE fractions and DIOHBA, we suggest that future studies DIOHBA quantification (post cupric oxidation) with DOM optical measurements. With additional validation, variation in spectral shape or in emission maxima under varying conditions such as pH could serve as a proxy for DOM fluorophores linked to DIOHBA precursors.

#### Structural and Compositional Controls on ^1^O_2_ and ^**•**^OH Formation

Correlations of ^1^O_2_ and ^•^OH formation with optical surrogates indicate that SRNOM fractions behave more consistently with the broader DOM literature, including ultrafiltration studies, than the above discussion would predict. For example, although E2:E3 and Φ_*f*_ do not vary systematically with electrophoretic mobility, Φ_Δ_ and Φ_OH_ are both well correlated to these parameters, consistent with a pattern frequently observed for ultrafiltered DOM,^13,15,24,80^ whole-water samples,^81,82^ and ozone-treated DOM (Figure 6).^83^ This suggests an overlap between the structural features governing E2:E3 and the excited states that are precursors to Φ_*f*_, Φ_Δ_, and Φ_OH_. Dalrymple et al. suggested that E2:E3 is inversely related to DOM’s ability to produce charge-transfer states.^84^ These states would serve as a radiationless deactivation pathway for ^1^DOM*, explaining the decrease in Φ_*f*_, Φ_Δ_, and Φ_OH_ with decreasing E2:E3. Additional research such as measuring spectra in varying solvent polarities or sodium borohydride reduction could further delineate the role of charge transfer in PFFE fractions. Regardless of the mechanism, these results indicate that the photophysical processes funneling excited states away from fluorescence are also funneling excited states away from triplet DOM (^1^O_2_ precursor) and ^•^OH precursors.

**Figure 6:**
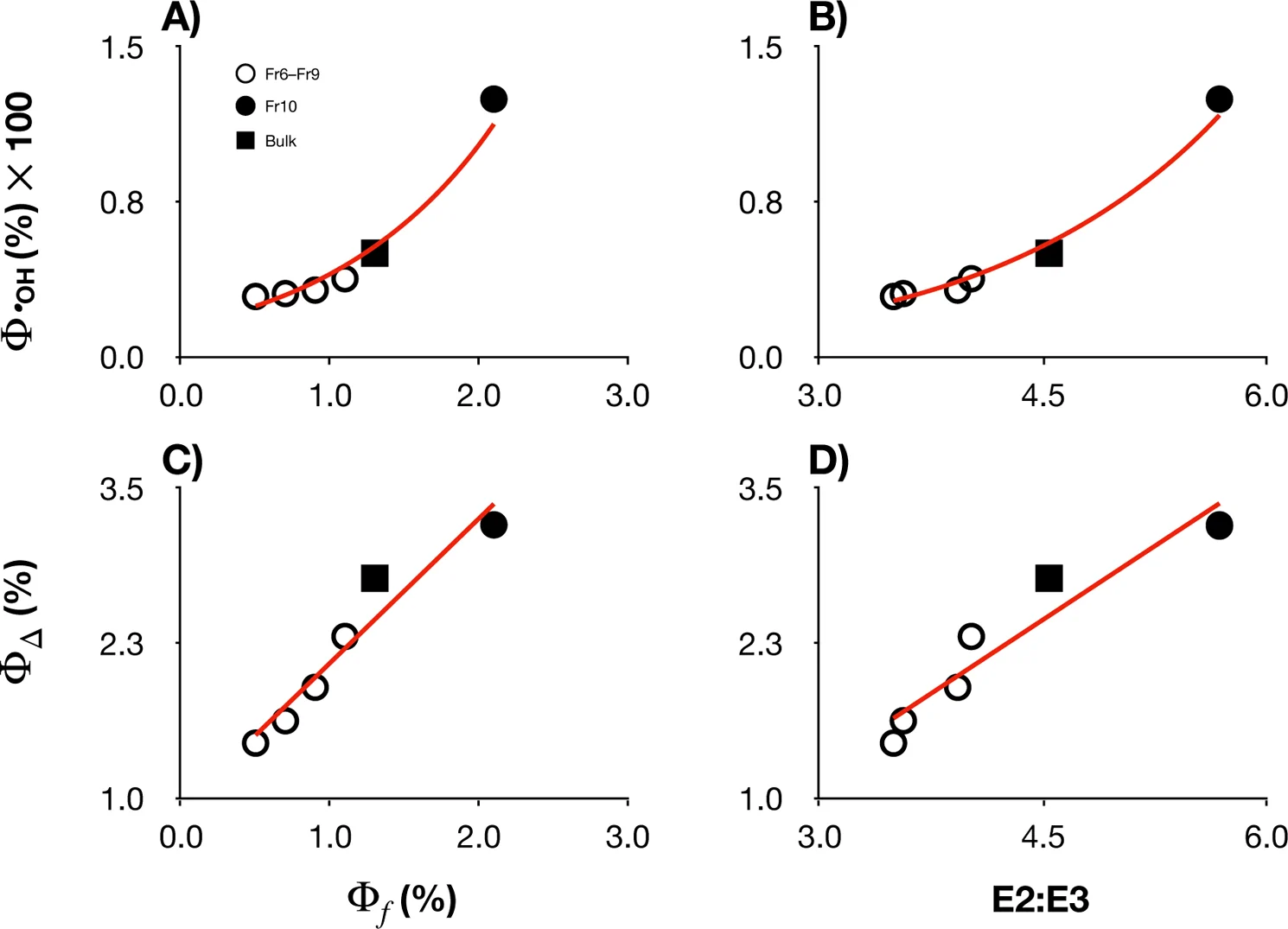
Quantum yields for the formation of (A and B) hydroxyl radical and (C and D) singlet oxygen in relation to fluorescence quantum yield (Φ_*f*_) and the E2:E3 ratio. Closed circles represent Fr10, closed squares represent bulk SRNOM, and open symbols represent Fr6–Fr9. Irradiation conditions: 12 365 nm bulbs in a Rayonet photoreactor with temperature ∼30 °C; [furfuryl alcohol] = 25 μM or [benzoic acid] = 2.0 mM; pH 7.2. Solid lines represent an exponential (A and B) or linear fit (C and D) to the data.

## Environmental Implications

In this work, we have shown that separation of SRNOM by PFFE results in structurally distinct fractions with optical and photochemical properties different from those of apparent molecular weight fractions generated by ultrafiltration. Moreover, the relationships between absorbance-based optical surrogates and compositional parameters (i.e., molecular weight and aromaticity) in some cases go against classical interpretations in the literature.^58^ The molecular size and aromaticity of Fr6 through Fr9 vary substantially, yet the E2:E3 values of these fractions are highly similar and their SUVA_254_ values do not vary systematically. Practically, this suggests that caution should be used when interpreting changes in optical properties in relation to structure for natural or engineered processes that fractionate DOM on the basis of charge density. At a fundamental level, these results provide evidence that additional structural descriptors (beyond aromaticity and molecular size) are necessary to fully understand E2:E3 and perhaps SUVA_254_.

We have also shown that DIOHBA precursors are strongly correlated with DOM optical properties, especially fluorescence. DIOHBA is believed to be linked to tannins and flavonoids and not lignin. Although tannins have been hypothesized to be a source of chromophoric DOM,^85–87^ there is, to our knowledge, very little direct evidence for their role, in stark contrast to the multitude of studies showing correlations between absorbance and lignin phenol concentrations.^64,65^ Future studies should employ cupric oxide oxidation and DIOHBA quantification in environmental field studies as well as mechanistic investigations to further understand the role of DIOHBA precursors in DOM optical properties.

Despite the atypical behavior of absorbance-based surrogates for PFFE fractions, correlations between photochemical quantum yields and optical surrogates (i.e., E2:E3 and Φ_*f*_) hold across the fractions in a manner typical of ultrafiltration-generated fractions. This points to E2:E3 being a predictor of reactivity that cannot be ascribed to something as simple as molecular weight alone (e.g., the abundance and nature of interchromophore interactions).

On the basis of its unique results compared to ultrafiltration, PFFE has shown utility in separating DOM, which remains an outstanding analytical challenge to characterize.^88^ The in-depth analysis of PFFE-generated fractions presented herein is an exemplary top-down approach for tackling this challenge.^35,44^ PFFE offers the fundamental advantage of avoiding interactions with a solid-phase matrix. In addition, electrophoretic separation is based not only on size but also on charge density, which more directly relates the separation process to DOM structure and, therefore, the mechanistic role of DOM in natural and engineered aquatic systems. This is an advantage over ultrafiltration methods, which function primarily based on size. Drawbacks of PFFE are that the separation device itself is an instrument with higher cost than the materials typically used for DOM fractionation, such as ultrafiltration cells and membranes or solid-phase extraction cartridges. Other disadvantages are the need to separate the resulting fractions from the aqueous buffer employed in the separation medium and the comparatively low throughput compared with solid-phase extraction or ultrafiltration.

It is worth considering PFFE for the separation of additional DOM isolates readily available in high quantities (e.g., IHSS isolates). For example, it would be of interest to see if the trends observed for SRNOM are different for fulvic acid extracts (which have higher carboxyl group abundance than SRNOM).^39^ Furthermore, investigating the behavior of PFFE fractions in mineral sorption, microbial processing, organic pollutant and metal interactions, and photomineralization is another productive research avenue, given that DOM size and charge are known to influence each of these processes. For example, one could quantify the metal-binding constants of PFFE fractions and compare them to the bulk sample with a conservative mixing model. Finally, DOM is one of many complex carbon-based mixtures^89^ for which structure–reactivity relationships remain elusive, suggesting that PFFE may also prove to be a powerful separation tool beyond the DOM field.

## Supplementary Material



## Notes

Experimental and analytical methods, details regarding the analysis of data, and additional data and figures (PDF)

## References

[1] Kieber, D. J. ; Powers, L. C. ; Stubbins, A. ; Miller, W. L. Marine photochemistry of organic matter: Processes and impacts. In Biogeochemistry of Marine Dissolved Organic Matter; 3rd ed.; Hansell, D.A., Carlson, C.A., Eds., Academic Press: London, 2024; pp. 507–585.DOI:10.1016/B978-0-443-13858-4.00009-5.

[2] Bushaw K. L., Zepp R. G., Tarr M. A., Schulz-Jander D., Bourbonniere R. A., Hodson R. E., Miller W. L., Bronk D. A., Moran M. A. (1996). Photochemical Release of Biologically
Available Nitrogen
from Aquatic Dissolved Organic Matter. Nature.

[3] Vähätalo A. V., Zepp R. G. (2005). Photochemical Mineralization of Dissolved Organic Nitrogen
to Ammonium in the Baltic Sea. Environ. Sci.
Technol..

[4] Ossola R., Gruseck R., Houska J., Manfrin A., Vallieres M., McNeill K. (2022). Photochemical Production of Carbon Monoxide from Dissolved
Organic Matter: Role of Lignin Methoxyarene Functional Groups. Environ. Sci. Technol..

[5] Ossola R., Tolu J., Clerc B., Erickson P. R., Winkel L. H. E., McNeill K. (2019). Photochemical Production
of Sulfate and Methanesulfonic
Acid from Dissolved Organic Sulfur. Environ.
Sci. Technol..

[6] Packer J. L., Werner J. J., Latch D. E., McNeill K., Arnold W. A. (2003). Photochemical
Fate of Pharmaceuticals in the Environment: Naproxen, Diclofenac,
Clofibric Acid, and Ibuprofen. Aquat. Sci..

[7] Latch D. E., Stender B. L., Packer J. L., Arnold W. A., McNeill K. (2003). Photochemical
Fate of Pharmaceuticals in the Environment: Cimetidine and Ranitidine. Environ. Sci. Technol..

[8] Bodrato M., Vione D. (2014). APEX (Aqueous Photochemistry
of Environmentally Occurring Xenobiotics):
A Free Software Tool to Predict the Kinetics of Photochemical Processes
in Surface Waters. Environ. Sci.: Processes
Impacts.

[9] Mena C., Reglero P., Hidalgo M., Sintes E., Santiago R., Martín M., Moyà G., Balbín R. (2019). Phytoplankton
Community Structure Is Driven by Stratification in the Oligotrophic
Mediterranean Sea. Front. Microbiol..

[10] Hickman A., Dutkiewicz S., Williams R., Follows M. (2010). Modelling the Effects
of Chromatic Adaptation on Phytoplankton Community Structure in the
Oligotrophic Ocean. Mar. Ecol.: Prog. Ser..

[11] Buckley S., McKay G., Leresche F., Rosario-Ortiz F. (2024). Inferring
the Molecular Basis for Dissolved Organic Matter Photochemical and
Optical Properties. Environ. Sci. Technol..

[12] Chin Y.-P., Aiken G., O’Loughlin E. (1994). Molecular Weight, Polydispersity,
and Spectroscopic Properties of Aquatic Humic Substances. Environ. Sci. Technol..

[13] Maizel A. C., Remucal C. K. (2017). Molecular Composition and Photochemical Reactivity
of Size-Fractionated Dissolved Organic Matter. Environ. Sci. Technol..

[14] Cory R. M., Kling G. W. (2018). Interactions between
Sunlight and Microorganisms Influence
Dissolved Organic Matter Degradation along the Aquatic Continuum. Limnology Oceanogr Lett..

[15] McKay G., Couch K. D., Mezyk S. P., Rosario-Ortiz F. L. (2016). Investigation
of the Coupled Effects of Molecular Weight and Charge-Transfer Interactions
on the Optical and Photochemical Properties of Dissolved Organic Matter. Environ. Sci. Technol..

[16] Shimabuku K. K., Kennedy A. M., Mulhern R. E., Summers R. S. (2017). Evaluating Activated
Carbon Adsorption of Dissolved Organic Matter and Micropollutants
Using Fluorescence Spectroscopy. Environ. Sci.
Technol..

[17] Buckley S., Leresche F., Hanson B., Rosario-Ortiz F. L. (2023). Decoupling
Optical Response and Photochemical Formation of Singlet Oxygen in
Size Isolated Fractions of Ozonated Dissolved Organic Matter. Environ. Sci. Technol..

[18] Ward C. P., Sleighter R. L., Hatcher P. G., Cory R. M. (2014). Insights into the
Complete and Partial Photooxidation of Black Carbon in Surface Waters. Environ. Sci.: Processes Impacts.

[19] Schmitt-Kopplin P., Hertkorn N., Schulten H.-R., Kettrup A. (1998). Structural Changes
in a Dissolved Soil Humic Acid during Photochemical Degradation Processes
under O2 and N2 Atmosphere. Environ. Sci. Technol..

[20] Wert E. C., Gonzales S., Dong M. M., Rosario-Ortiz F. L. (2011). Evaluation
of Enhanced Coagulation Pretreatment to Improve Ozone Oxidation Efficiency
in Wastewater. Water Res..

[21] Perminova, I. V. ; Konstantinov, A. I. ; Kunenkov, E. V. ; Gaspar, A. ; Schmitt-Kopplin, P. ; Hertkorn, N. ; Kulikova, N. A. ; Hatfield, K. Separation technology as a powerful tool for unfolding molecular complexity of natural organic matter and humic substances. In Biophysico-Chemical Processes Involving Natural Nonliving Organic Matter In Environmental Systems; Huang, P.M., Senesi, N., Eds.Wiley: Hoboken, NJ 2009, pp. 487–538 ; DOI: 10.1002/9780470494950

[22] Peuravuori J., Pihlaja K. (2004). Preliminary Study of
Lake Dissolved Organic Matter
in Light of Nanoscale Supramolecular Assembly. Environ. Sci. Technol..

[23] McAdams B. C., Aiken G. R., McKnight D. M., Arnold W. A., Chin Y.-P. (2018). High Pressure
Size Exclusion Chromatography (HPSEC). Determination of Dissolved
Organic Matter Molecular Weight Revisited: Accounting for Changes
in Stationary Phases, Analytical Standards, and Isolation Methods. Environ. Sci. Technol..

[24] Mostafa, S. ; Korak, J. A. ; Shimabuku, K. ; Glover, C. M. ; Rosario-Ortiz, F. L. Relation between Optical Properties and Formation of Reactive Intermediates from Different Size Fractions of Organic Matter. In Advances in the Physicochemical Characterization of Dissolved Organic Matter: Impact on Natural and Engineered Systems ; Rosario-Ortiz, F.L., Ed; American Chemical Society, 2014, Vol. 1160, 2014 1160 159–179 10.1021/bk-2014-1160.ch008

[25] Trubetskoj O. A., Richard C., Voyard G., Marchenkov V. V., Trubetskaya O. E. (2018). Molecular Size Distribution of Fluorophores
in Aquatic
Natural Organic Matter: Application of HPSEC with Multi-Wavelength
Absorption and Fluorescence Detection Following LPSEC-PAGE Fractionation. Environ. Sci. Technol..

[26] Guéguen C., Cuss C. W. (2011). Characterization
of Aquatic Dissolved Organic Matter
by Asymmetrical Flow Field-Flow Fractionation Coupled to UV–Visible
Diode Array and Excitation Emission Matrix Fluorescence. J. Chromatogr. A.

[27] Xue J.-P., Cuss C. W., Wang Y., Javed M. B., Noernberg T., Pelletier R., Shotyk W. (2022). Size-Resolved Fluorescence Underscores
Negligible Interaction of Dissolved Organic Matter During Conservative
Mixing in a Large Boreal River. Front. Environ.
Chem..

[28] Haan H. D., Boer T. D. (1987). Applicability of
Light Absorbance and Fluorescence
as Measures of Concentration and Molecular Size of Dissolved Organic
Carbon in Humic Lake Tjeukemeer. Water Res..

[29] Piccolo A. (2001). The Supramolecular
Structure of Humic Substances. Soil Sci..

[30] Lan T., Wu P., Liu Z., Stroet M., Liao J., Chai Z., Mark A., Liu N., Wang D. (2022). Understanding the Effect
of pH on the Solubility and Aggregation Extent of Humic Acid in Solution
by Combining Simulation and the Experiment. Environ. Sci. Technol..

[31] Peuravuori J., Pihlaja K. (1997). Molecular Size Distribution
and Spectroscopic Properties
of Aquatic Humic Substances. Anal. Chim. Acta.

[32] Helms J. R., Stubbins A., Ritchie J. D., Minor E. C., Kieber D. J., Mopper K. (2008). Absorption Spectral
Slopes and Slope Ratios as Indicators
of Molecular Weight, Source, and Photobleaching of Chromophoric Dissolved
Organic Matter. Limnol. Oceanogr..

[33] Zherebker A., Shirshin E., Rubekina A., Kharybin O., Kononikhin A., Kulikova N. A., Zaitsev K. V., Roznyatovsky V. A., Grishin Y. K., Perminova I. V., Nikolaev E. N. (2020). Optical Properties
of Soil Dissolved Organic Matter Are Related to Acidic Functions of
Its Components as Revealed by Fractionation, Selective Deuteromethylation,
and Ultrahigh Resolution Mass Spectrometry. Environ. Sci. Technol..

[34] Wünsch U. J., Murphy K. (2021). A Simple Method to
Isolate Fluorescence Spectra from
Small Dissolved Organic Matter Datasets. Water
Res..

[35] Li Y., Harir M., Lucio M., Gonsior M., Koch B. P., Schmitt-Kopplin P., Hertkorn N. (2016). Comprehensive Structure-Selective
Characterization of Dissolved Organic Matter by Reducing Molecular
Complexity and Increasing Analytical Dimensions. Water Res..

[36] Schmitt-Kopplin P., Junkers J. (2003). Capillary Zone Electrophoresis of Natural Organic Matter. J. Chromatogr. A.

[37] Trubetskoj O. A., Trubetskaya O. E., Richard C. (2009). Photochemical Activity and Fluorescence
of Electrophoretic Fractions of Aquatic Humic Matter. Water Res..

[38] Trubetskoj O. A., Trubetskaya O. E., Afanas’eva G.
V., Reznikova O. I., Saiz-Jimenez C. (1997). Polyacrylamide Gel Electrophoresis of Soil Humic Acid
Fractionated by Size-Exclusion Chromatography and Ultrafiltration. J. Chromatogr. A.

[39] Ritchie J. D., Perdue E. M. (2003). Proton-Binding Study
of Standard and Reference Fulvic
Acids, Humic Acids, and Natural Organic Matter. Geochim. Cosmochim. Acta.

[40] Schendorf T. M., Vecchio R. D., Bianca M., Blough N. V. (2019). Combined Effects
of pH and Borohydride Reduction on Optical Properties of Humic Substances
(HS): A Comparison of Optical Models. Environ.
Sci. Technol..

[41] Allen A., Cheng K., McKay G. (2023). Evaluating the pH-Dependence of DOM
Absorbance, Fluorescence, and Photochemical Production of Singlet
Oxygen. Environ. Sci.: Processes Impacts.

[42] Gáspár, A. Unravelling the Interactions of Boron with Natural Organic Matter (NOM). on a Molecular Level; Technical University of Munich, 2008.

[43] Schmitt-Kopplin P., Kettrup A. (2003). Capillary Electrophoresis – Electrospray Ionization
– Mass Spectrometry for the Characterization of Natural Organic
Matter: An Evaluation with Free Flow Electrophoresis-off-line Flow
Injection Electrospray Ionization-mass Spectrometry. Electrophoresis.

[44] Gaspar A., Harir M., Hertkorn N., Schmitt-Kopplin P. (2010). Preparative
Free-flow Electrophoretic Offline ESI-Fourier Transform Ion Cyclotron
Resonance/MS Analysis of Suwannee River Fulvic Acid. Electrophoresis.

[45] Junkers J., Schmitt-Kopplin P., Munch J., Kettrup A. (2002). Up-scaling Capillary
Zone Electrophoresis Separations of Polydisperse Anionic Polyelectrolytes
with Preparative Free-flow Electrophoresis Exemplified with a Soil
Fulvic Acid. Electrophoresis.

[46] Kothawala D. N., Murphy K. R., Stedmon C. A., Weyhenmeyer G. A., Tranvik L. J. (2013). Inner Filter Correction of Dissolved
Organic Matter
Fluorescence. Limnol. Oceanogr.: Methods.

[47] Murphy K. R., Stedmon C. A., Graeber D., Bro R. (2013). Fluorescence Spectroscopy
and Multi-Way Techniques. PARAFAC. Anal. Methods.

[48] Korak J. A., McKay G. (2024). Meta-Analysis of Optical
Surrogates for the Characterization of Dissolved
Organic Matter. Environ. Sci. Technol..

[49] Zhou Q., Cabaniss S. E., Maurice P. A. (2000). Considerations in the Use of High-Pressure
Size Exclusion Chromatography (HPSEC) for Determining Molecular Weights
of Aquatic Humic Substances. Water Res..

[50] Wünsch U. J., Stedmon C. A., Tranvik L. J., Guillemette F. (2018). Unraveling
the Size-dependent Optical Properties of Dissolved Organic Matter. Limnol. Oceanogr..

[51] Aiken G. R., Malcolm R. L. (1987). Molecular Weight
of Aquatic Fulvic Acids by Vapor Pressure
Osmometry. Geochim. Cosmochim. Acta.

[52] Kaiser K., Benner R. (2005). Hydrolysis-induced
Racemization of Amino Acids. Limnol Oceanogr
Methods.

[53] Cheng K., Li H., Laszakovits J. R., Sharpless C. M., Rosario-Ortiz F., McKay G. (2025). Probing the Photochemical Formation
of Hydroxyl Radical from Dissolved Organic Matter: Insights into the
H2O2-Dependent Pathway. Environ. Sci. Technol..

[54] Laszakovits J. R., Berg S. M., Anderson B. G., O’brien J. E., Wammer K. H., Sharpless C. (2017). M. p-Nitroanisole/Pyridine and p-Nitroacetophenone/Pyridine
Actinometers Revisited: Quantum Yield in Comparison to Ferrioxalate. Environ. Sci. Technol. Lett..

[55] McNeill K., Canonica S. (2016). Triplet State Dissolved
Organic Matter in Aquatic Photochemistry:
Reaction Mechanisms, Substrate Scope, and Photophysical Properties. Environ. Sci.: Processes Impacts.

[56] Partanen S. B., Erickson P. R., Latch D. E., Moor K. J., McNeill K. (2020). Dissolved
Organic Matter Singlet Oxygen Quantum Yields: Evaluation Using Time-Resolved
Singlet Oxygen Phosphorescence. Environ. Sci.
Technol..

[57] Leenheer, J. A. ; Malcolm, R. L. Preparative Free-Flow Electrophoresis as a Method of Fractionation of Natural Organic Materials; Water Supply Paper; USGS Numbered Series, Number 1817, 1973 10.3133/Wsp1817D.

[58] Korak J. A., McKay G. (2024). Critical Review of Fluorescence and Absorbance Measurements as Surrogates
for the Molecular Weight and Aromaticity of Dissolved Organic Matter. Environ. Sci.: Processes Impacts.

[59] Green S. A., Morel F. M. M., Blough N. V. (1992). Investigation
of the Electrostatic
Properties of Humic Substances by Fluorescence Quenching. Environ. Sci. Technol..

[60] Li H., McKay G. (2023). Fluorescence Quenching
of Humic Substances and Natural Organic Matter
by Nitroxide Free Radicals. Environ. Sci. Technol..

[61] Dryer D. J., Korshin G. V., Fabbricino M. (2008). In Situ Examination of the Protonation
Behavior of Fulvic Acids Using Differential Absorbance Spectroscopy. Environ. Sci. Technol..

[62] Gabor, R. ; Baker, A. ; McKnight, D. M. ; Miller, M. P. Fluorescence Indices and Their Interpretation. In Aquatic Organic Matter Fluorescence; Coble, P. G. ; Lead, J. ; Baker, A. ; Reynolds, D. M. ; Spencer, R. G. M. eds., 2014; pp. 303–338. 10.1017/CBO9781139045452.015.

[63] Weishaar J. L., Aiken G. R., Bergamaschi B. A., Fram M. S., Fujii R., Mopper K. (2003). Evaluation of Specific
Ultraviolet Absorbance as an
Indicator of the Chemical Composition and Reactivity of Dissolved
Organic Carbon. Environ. Sci. Technol..

[64] Fichot C.
G., Benner R. (2012). The Spectral
Slope Coefficient of Chromophoric Dissolved
Organic Matter (S275–295). as a Tracer of Terrigenous Dissolved
Organic Carbon in River-influenced Ocean Margins. Limnol. Oceanogr..

[65] Fichot C. G., Benner R., Kaiser K., Shen Y., Amon R. M. W., Ogawa H., Lu C.-J. (2016). Predicting
Dissolved Lignin Phenol
Concentrations in the Coastal Ocean from Chromophoric Dissolved Organic
Matter (CDOM). Absorption Coefficients. Front.
Mar. Sci..

[66] Dickens A. F., Gudeman J. A., Gélinas Y., Baldock J. A., Tinner W., Hu F. S., Hedges J. I. (2007). Sources
and Distribution of CuO-Derived
Benzene Carboxylic Acids in Soils and Sediments. Org. Geochem..

[67] Yamashita Y., Fichot C. G., Shen Y., Jaffé R., Benner R. (2015). Linkages among Fluorescent Dissolved
Organic Matter,
Dissolved Amino Acids and Lignin-Derived Phenols in a River-Influenced
Ocean Margin. Front. Marine Sci..

[68] Berg S. M., Wammer K. H., Remucal C. K. (2023). Dissolved
Organic Matter Photoreactivity
Is Determined by Its Optical Properties, Redox Activity, and Molecular
Composition. Environ. Sci. Technol..

[69] Richard C., Trubetskaya O., Trubetskoj O., Reznikova O., Afanas’eva G., Aguer J.-P., Guyot G. (2004). Key Role of the Low
Molecular Size Fraction of Soil Humic Acids for Fluorescence and Photoinductive
Activity. Environ. Sci. Technol..

[70] McKnight D.
M., Boyer E. W., Westerhoff P. K., Doran P. T., Kulbe T., Andersen D. T. (2001). Spectrofluorometric
Characterization of Dissolved Organic
Matter for Indication of Precursor Organic Material and Aromaticity. Limnol. Oceanogr..

[71] Huguet A., Vacher L., Relexans S., Saubusse S., Froidefond J., Parlanti E. (2009). Properties of Fluorescent
Dissolved Organic Matter
in the Gironde Estuary. Org. Geochem..

[72] Kellerman A. M., Guillemette F., Podgorski D. C., Aiken G. R., Butler K. D., Spencer R. G. M. (2018). Unifying Concepts Linking Dissolved Organic Matter
Composition to Persistence in Aquatic Ecosystems. Environ. Sci. Technol..

[73] Korak J. A., Dotson A. D., Summers R. S., Rosario-Ortiz F. L. (2014). Critical
Analysis of Commonly Used Fluorescence Metrics to Characterize Dissolved
Organic Matter. Water Res..

[74] Turro, N. J. ; Ramamurthy, V. ; Scaiano, J. C. Modern Molecular Photochemistry of Organic Molecules; University Science Books; University Science Books, 2012.

[75] Hernes P. J., Bergamaschi B. A., Eckard R. S., Spencer R. G. M. (2009). Fluorescence-based
Proxies for Lignin in Freshwater Dissolved Organic Matter. J. Geophys. Res. Biogeosci..

[76] Spencer R. G. M., Aiken G. R., Wickland K. P., Striegl R. G., Hernes P. J. (2008). Seasonal
and Spatial Variability in Dissolved Organic Matter Quantity and Composition
from the Yukon River Basin, Alaska. Glob. Biogeochem.
Cycles.

[77] Gandois L., Teisserenc R., Cobb A. R., Chieng H. I., Lim L. B. L., Kamariah A. S., Hoyt A., Harvey C. F. (2014). Origin, Composition,
and Transformation of Dissolved Organic Matter in Tropical Peatlands. Geochim. Cosmochim. Acta.

[78] Boyle E. S., Guerriero N., Thiallet A., Vecchio R. D., Blough N. V. (2009). Optical
Properties of Humic Substances and CDOM: Relation to Structure. Environ. Sci. Technol..

[79] Forest K., Wan P., Preston C. M. (2004). Catechin
and Hydroxybenzhydrols as Models for the Environmental
Photochemistry of Tannins and Lignins. Photochem.
Photobiol. Sci..

[80] McCabe A. J., Arnold W. A. (2016). Seasonal and Spatial Variabilities in the Water Chemistry
of Prairie Pothole Wetlands Influence the Photoproduction of Reactive
Intermediates. Chemosphere.

[81] Peterson B. M., McNally A. M., Cory R. M., Thoemke J. D., Cotner J. B., McNeill K. (2012). Spatial and Temporal
Distribution of Singlet Oxygen
in Lake Superior. Environ. Sci. Technol..

[82] McKay G., Huang W., Romera-Castillo C., Crouch J. E., Rosario-Ortiz F. L., Jaffé R. (2017). Predicting
Reactive Intermediate Quantum Yields from
Dissolved Organic Matter Photolysis Using Optical Properties and Antioxidant
Capacity. Environ. Sci. Technol..

[83] Leresche F., McKay G., Kurtz T., Gunten U. V., Canonica S., Rosario-Ortiz F. L. (2019). Effects of Ozone on the Photochemical and Photophysical
Properties of Dissolved Organic Matter. Environ.
Sci. Technol..

[84] Dalrymple R. M., Carfagno A. K., Sharpless C. M. (2010). Correlations between Dissolved Organic
Matter Optical Properties and Quantum Yields of Singlet Oxygen and
Hydrogen Peroxide. Environ. Sci. Technol..

[85] Maie N., Parish K. J., Watanabe A., Knicker H., Benner R., Abe T., Kaiser K., Jaffé R. (2006). Chemical Characteristics of Dissolved
Organic Nitrogen in an Oligotrophic Subtropical Coastal Ecosystem. Geochim. Cosmochim. Acta.

[86] Sleighter R. L., Hatcher P. G. (2008). Molecular Characterization
of Dissolved Organic Matter
(DOM). along a River to Ocean Transect of the Lower Chesapeake Bay
by Ultrahigh Resolution Electrospray Ionization Fourier Transform
Ion Cyclotron Resonance Mass Spectrometry. Mar.
Chem..

[87] Andrew A. A., Vecchio R. D., Subramaniam A., Blough N. V. (2013). Chromophoric Dissolved
Organic Matter (CDOM) in the Equatorial Atlantic Ocean: Optical Properties
and Their Relation to CDOM Structure and Source. Mar. Chem..

[88] Li S., Harir M., Bastviken D., Schmitt-Kopplin P., Gonsior M., Enrich-Prast A., Valle J., Hertkorn N. (2024). Dearomatization
Drives Complexity Generation in Freshwater Organic Matter. Nature.

[89] Madhu M., Ilina A., Li H., McKay G., Kohler B. (2026). Common Photoproperties
of Eumelanin and Natural Organic Matter Emerge from Ensembles of Few-Layered
Nanostructures. ACS Cent. Sci..

