# Supplementary material for: Optical and Photochemical Properties of Suwannee River Natural Organic Matter Fractions Generated by Preparative Free Flow Electrophoresis
Source: Environ Sci Technol. 2026 Sep 25;60(39):27829–40. doi: 10.1021/acs.est.6c07200 (PMC13648227; doi:10.1021/acs.est.6c07200)
Supplement: Supplementary file 1 [file es6c07200_si_001.pdf]

Supporting Information for

Optical and Photochemical Properties of Suwannee River Natural Organic Matter Fractions Generated by Preparative Free Flow Electrophoresis

Garrett McKay[a*], Kai Cheng[a], András Gáspár[b], Mourad Harir[b,c], Philippe Schmitt-Kopplin[b,c*], Norbert Hertkorn[b,d], Ronald Benner[e], Karl Kaiser[f,g*]

[a]Zachry Department of Civil & Environmental Engineering, Texas A&M University, College Station, TX 77843, United States

[b]Research Unit Analytical Biogeochemistry, Helmholtz Munich, 85764 Neuherberg, Germany

[c]Technical University of Munich, Chair of Analytical Food Chemistry, 85354 Freising, Germany

[d]Department of Thematic Studies−Environmental Change, Linköping University, SE 581 83 Linköping, Sweden 2455

[e]Department of Biological Sciences, University of South Carolina, Columbia, SC 29208 USA

[f]Department of Marine and Coastal Environmental Sciences, Texas A&M University, Galveston, Texas 77554, United States

[g]Department of Oceanography, Texas A&M University, College Station, Texas 77843, United States

***Corresponding authors**

Garrett McKay (email: gmckay@tamu.edu), Philippe Schmitt-Kopplin (email: philippe.schmittkopplin@helmholtz-munich.de), Karl Kaiser (email: kaiserk@tamug.edu)

Table of Tables

Table S1. Conservative mixing model results for selected signals. 7
Table S2. Mass balance for SRNOM and PFFE fractions. The mass of organic carbon (OC) was obtained from the product of the measured C weght % and the mass of each fraction. 7
Table S3.^{13}C-NMR functional group percentages for bulk SRNOM and fractions. 8
Table S4. Optical surrogates of bulk SRNOM and fractions 8
Table S5. Molecular weight and quantum yields of bulk SRNOM and fractions. 8
Table S6. Selected lignin phenol data for SRNOM fractions. Quantities are in μg mg^{-1}..8

Table of Figures

Figure S1. Electrophoretogram of SRNOM with absorbance detection. Vertical lines show the spacing of vials for collection of Fr1 to Fr11. 9
Figure S2. (Top) Heatmap of Spearman rank correlation coefficients for the relationship between optical/photochemical properties and compositional parameters. 10
Figure S3. Scatter plot of DIOHBA versus β/α for the SRNOM fractions. 10
Figure S4. Correlation between FI emission maxima. 11
Figure S5. Scatter plots of hydroxyl radical and singlet oxygen quantum yields versus aromaticity, M_n, and carbonyl content. 11
Figure S6. Emission spectra for Fr6–10 at (left) Ex=310 nm and (right) Ex=370 nm. ...12
Figure S7. HP-SEC chromatograms for Fr6-Fr10 and bulk SRNOM. 12
Figure S8. Relationship between singlet oxygen and hydroxyl radical quantum yields. 12
Figure S9. Electropherogram of SRNOM with absorbance at 254 nm detection. Right y-axis shows the amount in μmol of DIOHBA (3,5-dihydroxybenzoic acid) and $\Sigma6$...........13
Figure S10. Intensity weighted emission as a function of pH for bulk SRNOM, Fr6 and Fr9...14

1 Text S1. Analytical Methods

1.1 NMR[1]

A Bruker DMX spectrometer (B_0 = 11.7 T), with a cryogenic classical geometry 5 mm *z*-gradient cryogenic ^{13}C, ^{1}H probe and TopSpin 2.1 software were used to acquire ^{13}C NMR spectra of re-dissolved solid PFFE fractions of SRNOM (5–35 mg) in typically 75–135 µl CD_3OD (99.96% ^{2}H; CD_3OD; Merck, Darmstadt, Germany) at 283 K. Briefly, the re-dissolved DOM were transferred to 2.5–3.0-mm Bruker Match tubes and sealed. Transmitter pulses were at ~10 µs for ^{1}H and ^{13}C and calibrated 90°/180° pulses were used for each sample. ^{13}C NMR section integrals were computed by TopSpin 2.1 software. Functional group shift regions are provided in SI Table S2 and were 187-220 ppm (carbonyl), 167-187 ppm (carboxyl), 145-167 ppm (phenolics), 108-145 ppm (aromatics), 90-108 ppm (carbohydrate), 47-90 ppm (heteroaliphatic carbon), and 0-47 ppm (aliphatic carbon). We used inverse-gated ^{1}H decoupling for ^{13}C NMR spectra to eliminate nuclear Overhauser effects, with acquisition time aq = 1 s and relaxation delay d1 = 19 s.

1.2 Amino acid analysis[2]

Enantiomeric amino acids were separated on a Licrospher 100 RP18 (250×4 mm, 5 µm) with a guard column after in-line derivatization with *o*-phthaldialdehyde/*N*-isobutyryl-L-cysteine (OPA/IBLC) or *o*-phthaldialdehyde/*N*-isobutyryl-D-cysteine (OPA/IBDC) at 20 ºC. The system consisted of an Agilent HP 1100 with an autosampler and fluorescence detector. Samples were hydrolyzed with 6 M HCl in sealed ampoules for 20 h at 110 ºC. After hydrolysis, dry and neutral samples were dissolved in 100 µL water and 5 µL of L-glutamic acid-methylester, the internal standard, was added. Vigorous vortexing typically dissolved the entire dried residue in the micro-inserts. Insoluble particles were removed by centrifugation and the sample was carefully transferred to a new micro-insert if necessary. The mobile phases consisted of Phase A (40 mM KH_2PO_4 at pH 12) and Phase B (methanol/acetonitrile at 13:1 v/v). The linear gradient was as follows: 100% Phase A to 61% A at 50 min, 46% A at 72 min, 40% A at 80 min, then 100% A at 85 min, with a total run time of 87 min. The flow rate was set at 0.9 mL/min. Excitation was set at 350 nm and emission of the OPA derivatives were recorded at 420 nm.

1.3 Lignin analysis[3]

CuO oxidation was performed in heated steel vessels. After separation of unreacted CuO from mixtures by centrifugation, samples were extracted at pH 2 with Oasis HLB cartridges pre-conditioned with methanol and deionized water. After rinsing with water/methanol, cartridges were dried under vacuum and eluted sequentially with mixtures of dichloromethane/methylacetate/pyridine followed by two rinses of methanol. Dried extracts were analyzed by GC/MS according to Kaiser and Benner (2012).[3]

1.4 Optical measurements and calculations[4,5]

Absorbance and fluorescence spectra were measured on an Aqualog (Horiba). Data treatment included blank subtraction, inner filter corrections, removal of Rayleigh and Raman scatter, and normalization to the water Raman scattering area at a 350 nm excitation wavelength. The sum of optical densities at the excitation and emission wavelength ($A_{\lambda_{ex}} + A_{\lambda_{em}}$) was kept less than 1.5 to ensure appropriate inner filter corrections. Emission collection settings ranged from 0.1-4 s for the integration time, 2.33 nm increment, and a gain of medium. Optical surrogates and fluorescence quantum yields are calculated as described previously.[4,6,7]

Total organic carbon of filtered samples was measured with a Shimadzu V-SCH organic carbon analyzer.

1.5 Spectral pH titrations and fluorescence quenching[8,9]

Briefly, the pH of a ~25 mL aliquot was initially adjusted to 2.0 with 1 M HCl and then raised in 1-unit increments with 0.1 or 1 M NaOH up to pH 12. Sample was withdrawn at each integer pH, run on the Aqualog, and carefully returned to the beaker with a glass pipette. Using this method, the addition of titrant did not significantly impact the total solution volume (<5% total change).

Fluorescence quenching with 4-amino-TEMPO (tempamine) or 4-hydroxy-TEMPO (tempol) were performed as described previously.[10] The relative quenching by tempamine to tempol, quantified by the ratio of Stern-Volmer constants, is related to the pseudo electrostatic potential of DOM and thus serves as a metric for assessing the

change in fluorophore charge density[11] expected to occur in the PFFE samples. Quenching experiments were performed at pH 4 and 9 (buffered with acetate and carbonate, respectively) directly in fluorescence cuvettes via addition of small volumes of concentrated quencher. The precise concentration of the nitroxide quenchers were determined using the molar absorption coefficient at 434 nm (ε = 12.6 M^{-1} cm^{-1}).[11] Stern-Volmer plots for tempamine quenching exhibited substantial downward curvature, as noted previously.[10] Thus, quenching data for tempamine were fit with a two-component model whereby one group is inaccessible to quencher and the other is accessible.[10,12] Quenching plots for tempol were linear and therefore fit with the normal Stern-Volmer equation.

1.6 Size Exclusion Chromatography[13]

Size exclusion chromatography (SEC) was performed with an Ultimate 3000 UHPLC equipped with a Waters Protein-Pak 300SW column (7.5×300 mm, 10 µm particle size), which has lower and upper molecular weight ranges of 1,000 and 20,000, respectively, for random coil polymers. The mobile phase consisted of 0.1 M KCl and 5 mM total phosphate at pH 6.8. Injected samples were characterized by a photodiode array detector and fluorescence detector. Molecular weight was calibrated using polystyrene sulfonate (PSS) polymer standards prepared at 0.1 mg mL^{-1} with reported molecular weights ranging from 246 to 18,000 Da. The detection wavelength for calibration and DOM molecular weight determination was 222 nm, after McAdams et al.[13] The peak retention time of PSS standards was used for transforming sample's retention time into molecular weight. Thereafter, the number- and weight-average molecular weight were calculated according to

$$M_n = \Sigma_i h_i / \Sigma_i (h_i / M_i) \quad \text{Eq S 1}$$

$$M_w = \Sigma_i (h_i M_i) / \Sigma h_i \quad \text{Eq S 2}$$

where h_i represents the chromatogram signal (A_{222}) at molecular weight *i* (M_i). The high molecular weight limit was determined by multiplying the maximum h_i by 0.01, and the low molecular weight limit was set to 100 Da.[14]

1.7 Stern Volmer fitting

Our previous study[10] showed that fluorescence quenching by tempamine was best fit by a modified Stern-Volmer equation that assumes a fraction of fluorophores are inaccessible to quencher. Thus, tempamine quenching data were fit with the below equation

$$\int F^{f}_{Q,\lambda_{em}}\,d\lambda_{em} = \frac{f_a \int F^{f}_{0,\lambda_{em}}\,d\lambda_{em}}{1 + K_{SV}[Q]} + (1 - f_a)\int F^{f}_{0,\lambda_{em}}\,d\lambda_{em} \text{Eq}$$

where F^f is the fluorescence intensity in the presence and absence of quencher Q, f_a is the fraction of fluorophores that is accessible, and K_{SV} is the Stern-Volmer quenching constant of the accessible fraction. Tempol quenching data were fit with the linear Stern-Volmer equation $F^f_0/F^f_Q = 1 + K_{SV}[Q]$.

2 Text S2. Probe compound quantification and quantum yield analysis

Photochemistry methods. A Rayonet merry-go-round photoreactor was used for the photolysis reactions, consisting of a rotating sample holder, fan, and lamps emitting at a peak of 365 nm. The irradiance of the system was characterized using the *p*-nitroanisole (PNA)/pyridine actinometer (pyr), with [PNA] at 10 µM and [pyr] at 10 mM.[15] $^1O_2$ and $^{\bullet}OH$ were measured using the probe compounds furfuryl alcohol and benzoic acid, respectively, and quantum yields (Φ) were quantified as the rate of formation relative to the rate of light absorption. FFA photodegradation was monitored in SRNOM fraction solutions spiked with 25 µM FFA. The rate of $^1O_2$ formation was determined by dividing the FFA first-order photodegradation rate by the $^1O_2$-FFA bimolecular rate constant (1.0×10^8 $M^{-1}s^{-1}$)[16] and multiplying by the $^1O_2$ deactivation rate in water (2.5×10^5 s^{-1}). For $^{\bullet}OH$, 2 mM benzoate was employed to quantitatively scavenge all $^{\bullet}OH$ radicals. Dividing the rate of salicylic acid formation by the yield (0.204) results in the rate of $^{\bullet}OH$ formation. The rate of light absorption (R_a) was calculated by $\Sigma_\lambda I_{0,\lambda}\left(1 - 10^{-A_\lambda \ell}\right)$ where $I_{0,\lambda}$ is the irradiance, and $A_\lambda \ell$ is the product of the absorbance (cm^{-1}) and path length (0.84 cm). Quantum yields for each reactive intermediate (RI) were calculated relative to PNA/pyr considering the quantum yield for PNA photodegradation at [pyr]=10 mM ($\Phi_{RI} = \frac{R_{RI}}{R^{\circ}_{PNA}}\frac{R_{a,PNA}}{R_{a,DOM}}\Phi_{PNA}$). Note that R°_{PNA} is the initial degradation rate of *p*-nitroanisole obtained

by multiplying the initial concentration of 10 μM by the measured first-order photodegradation rate.

3 Text S3. Conservative mixing model

A conservative mixing model was employed to the SRNOM fraction data to assess for non-additivity. The model assumes that the weighted sum of observables for SRNOM fractions should add up to the bulk sample. The weighting factor can be expressed as the mass ratio obtained via PFFE on a total mass or mass of C basis

$$S_{bulk} = \sum_{i=1}^{5} f_i S_i \qquad \text{Eq S 4}$$

where S represents the signal of either the bulk SRNOM (S_{bulk}) or one of the five SRNOM fractions (S_i). f_i is mass ratio based on either total mass or mass of C.

Results from the conservative mixing model canulations are shown in Table S1.

Table S1. Conservative mixing model results for selected signals.

		Total mass		Mass of C	
	**Measured**	**Calculated**	**% Difference**	**Calculated**	**% Difference**
$SUVA_{254}$	4.38	2.99	-32	3.81	-13
FI	1.39	1.03	-26	1.30	-6
M_n	710	612	-14	780	10
Aromaticity	33.4	27.3	-18	34.8	5
λ_{pH4}	2.62	1.97	-25	2.50	-5
λ_{pH9}	5.19	3.04	-42	3.84	-26
$\Phi_{\Delta}\times10^2$	2.78	1.30	-47	1.65	-33
$\Phi_{OH}\times10^5$	5.04	2.69	-53	3.37	-41

4 Text S4. Additional data figures and tables

Table S2. Mass balance for SRNOM and PFFE fractions. The mass of organic carbon (OC) was obtained from the product of the measured C weght % and the mass of each fraction.

	**Wt % C**	**Mass obtained (mg)**	**Mass OC (mg)**
SRNOM	47.67	368.7	175.8
FR2	-	1.7	-
FR3	17.44	0.7	0.1
FR5	39.24	11.2	4.4
FR6	42.66	60.8	25.9
FR7	45.27	100.4	45.4
FR8	45.83	68.5	31.4
FR9	42.61	38.8	16.5
FR10	38.63	11.1	4.3

FR11	12.71	1.0	0.1
	**Sum (mg)**	294.1	128.2
	**Yield of fractionation**	80%	73%
	**% Fr6-Fr10**	95%	96%

Table S3. ^{13}C-NMR functional group percentages for bulk SRNOM and fractions.

**Fraction $\delta(^{13}C)$ [ppm]**	**Carbonyl 187-220**	**Carboxyl 167-187**	**Phenolics 145-167**	**Aromatic 108-145**	**Carbohydrate 90-108**	**Hetero C 47-90**	**Aliphatic C 0-47**
Fr6	4.1	19.6	11.4	25.5	9.3	17.7	12.6
Fr7	6.7	16.5	11.7	25.5	8.7	17.0	13.9
Fr8	3.6	12.4	12.2	27.0	9.7	23.4	11.7
Fr9	3.6	10.0	7.8	21.5	9.5	29.5	18.0
Fr10	1.8	11.9	6.4	18.4	7.3	31.7	22.5
Bulk	2.7	14.4	11.1	22.3	9.1	22.1	18.4

Table S4. Optical surrogates of bulk SRNOM and fractions

**Fraction**	**SUVA L mg_C^{-1} m^{-1}**	**$S_{275-295}$ μm^{-1}**	**$S_{350-400}$ μm^{-1}**	**E2:E3**	**FI**	β/α	**Φ_f x100**	**λ_{em}^{max}@ Ex=370 (nm)**	**λ_{em}^{max}@ Ex=310 (nm)**
Fr6	5.12	11.8	15.8	3.92	1.30	0.455	0.913	458	458
Fr7	3.20	11.3	14.6	3.49	1.29	0.480	0.540	456	456
Fr8	4.85	11.4	14.5	3.56	1.38	0.582	0.676	450	452
Fr9	3.29	12.0	14.6	4.02	1.51	0.640	1.052	448	448
Fr10	0.98	18.1	17.1	5.68	1.58	0.781	2.051	435	440
Bulk	4.38	13.1	16.6	4.54	1.39	0.477	1.264	456	454

Table S5. Molecular weight and quantum yields of bulk SRNOM and fractions.

	**Fr6**	**Fr7**	**Fr8**	**Fr9**	**Fr10**	**Bulk**
**Mn (Da)**	938	847	830	587	344	710
**Mw (Da)**	1912	2761	5519	4274	775	1444
**Φ_Δx10^2**	1.90	1.45	1.63	2.31	3.21	2.78
**Φ_{OH}x10^5**	3.26	2.95	3.09	3.81	12.5	5.04

Table S6. Selected lignin phenol data for SRNOM fractions. Quantities are in μg mg^{-1}.

**Fraction**	P	V	S	Σ_6	DIOHBA
Fr6	28.8	6.3	3.1	9.3	53.1
Fr7	55.7	23.5	11.7	35.2	44.9
Fr8	80.1	48.2	28.9	77.0	36.4
Fr9	68.5	34.0	23.5	57.4	20.7
Fr10	7.5	2.7	1.5	4.2	2.1

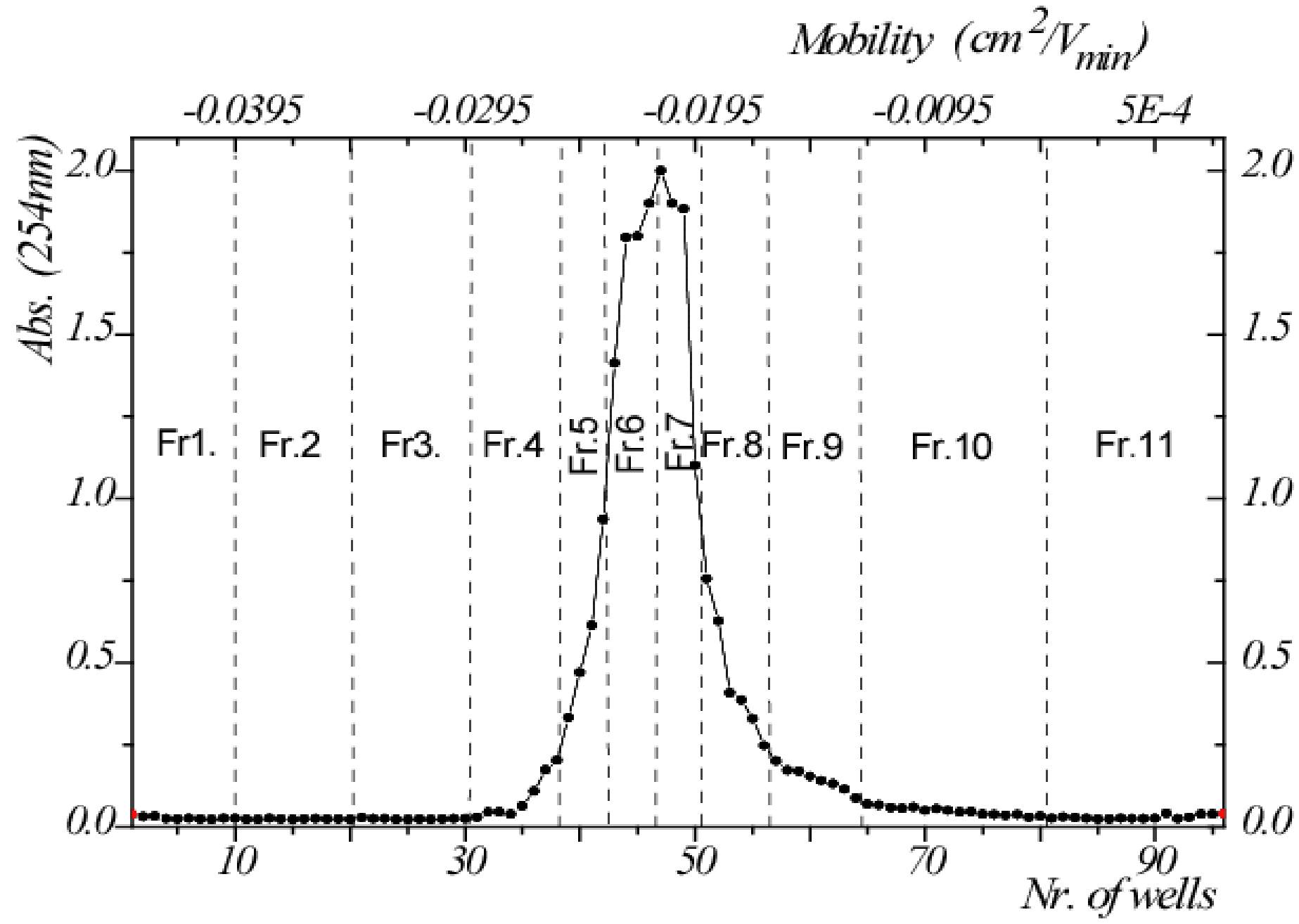

Figure S1. Electrophoretogram of SRNOM with absorbance detection. Vertical lines show the spacing of vials for collection of Fr1 to Fr11.

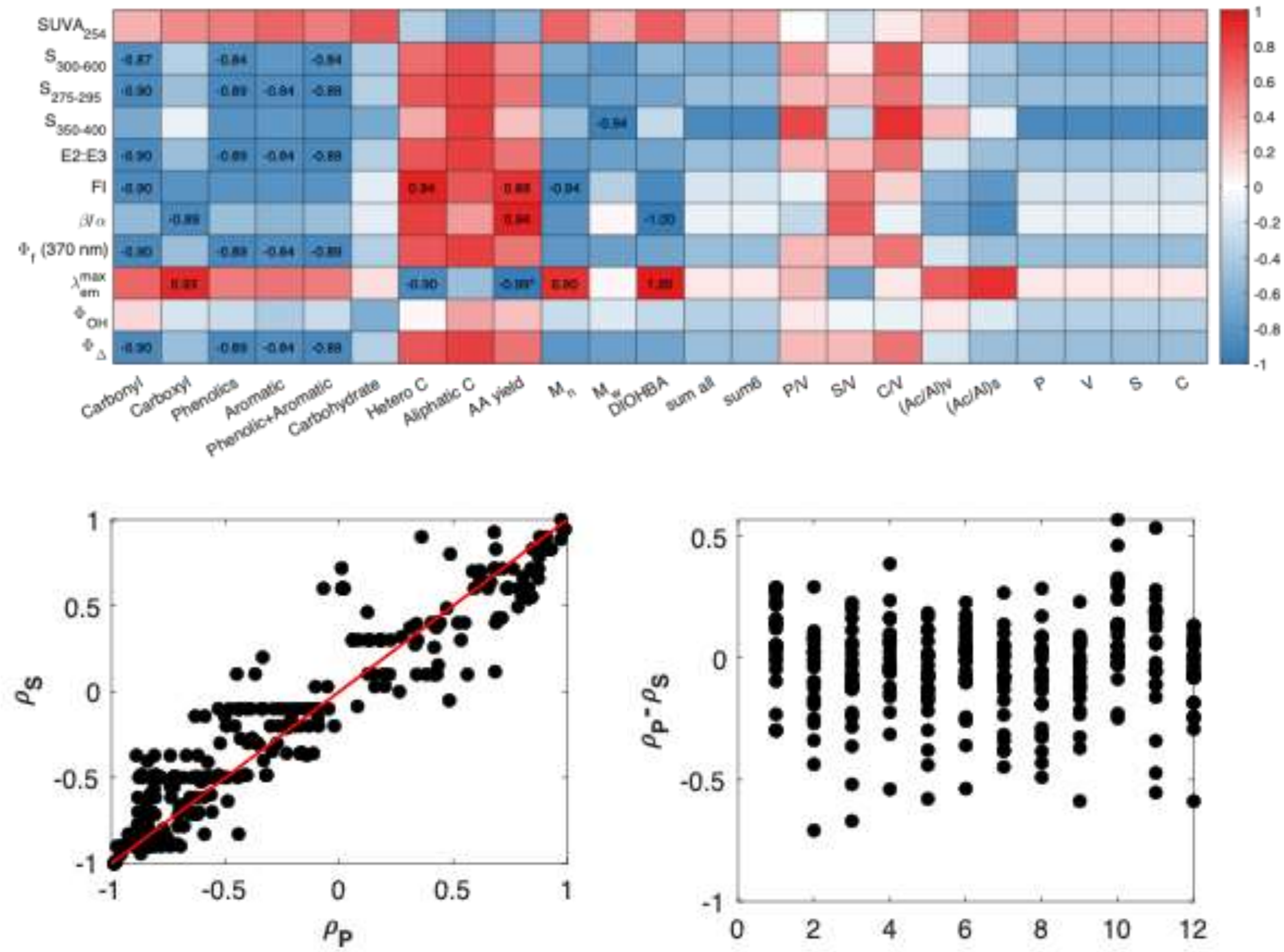

Figure S2. (Top) Heatmap of Spearman rank correlation coefficients for the relationship between optical/photochemical properties and compositional parameters. (Bottom) Spearman versus Pearson rho values and their difference.

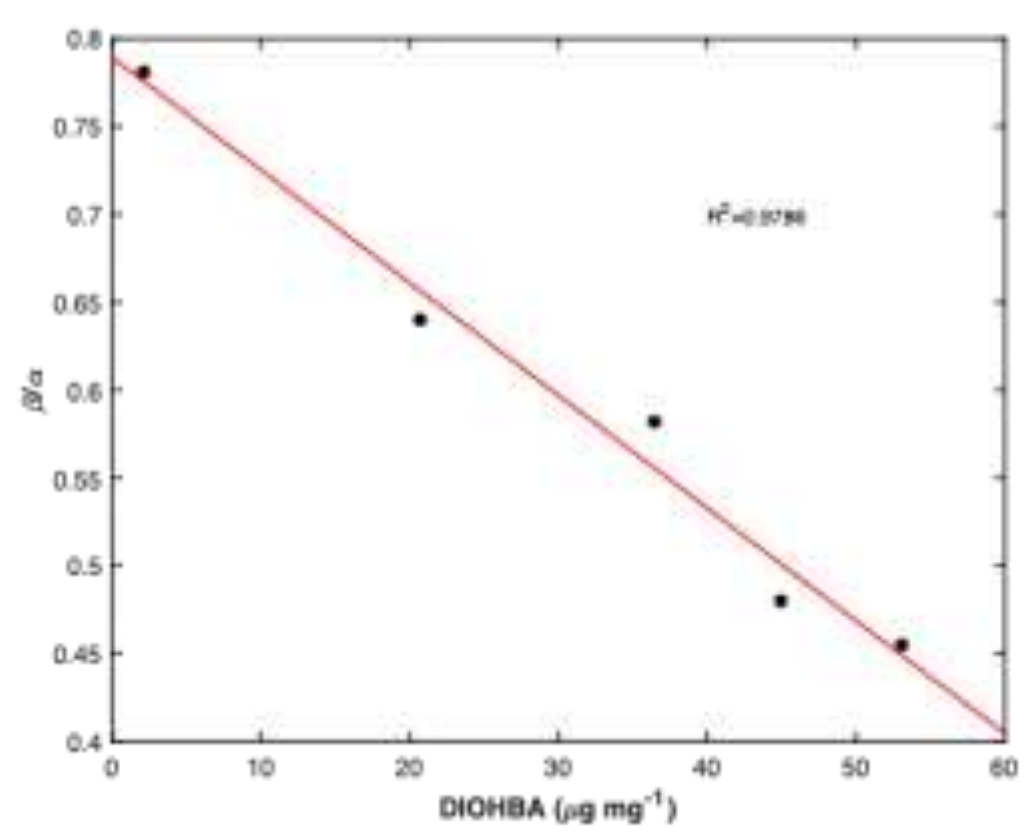

Figure S3. Scatter plot of DIOHBA versus β/α for the SRNOM fractions.

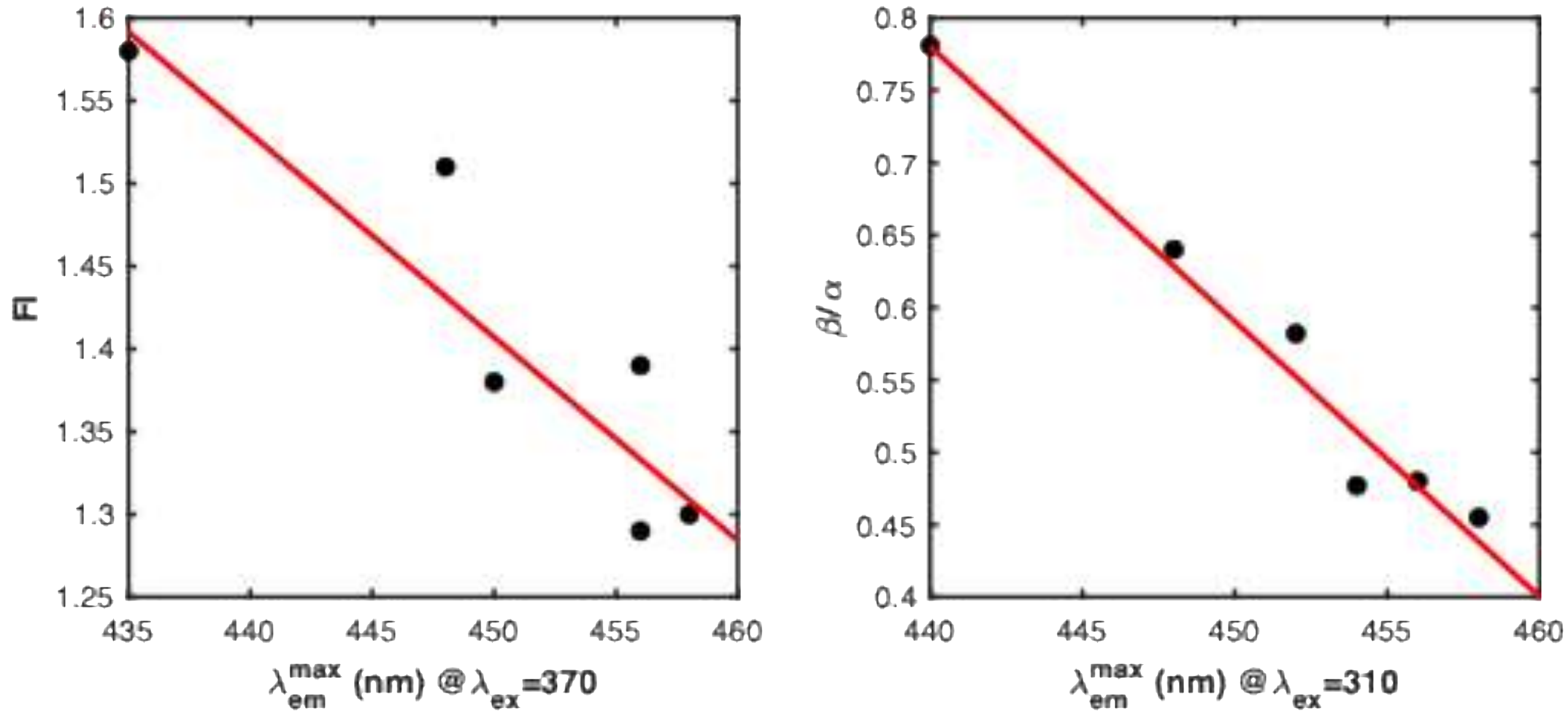

Figure S4. Correlation between FI emission maxima.

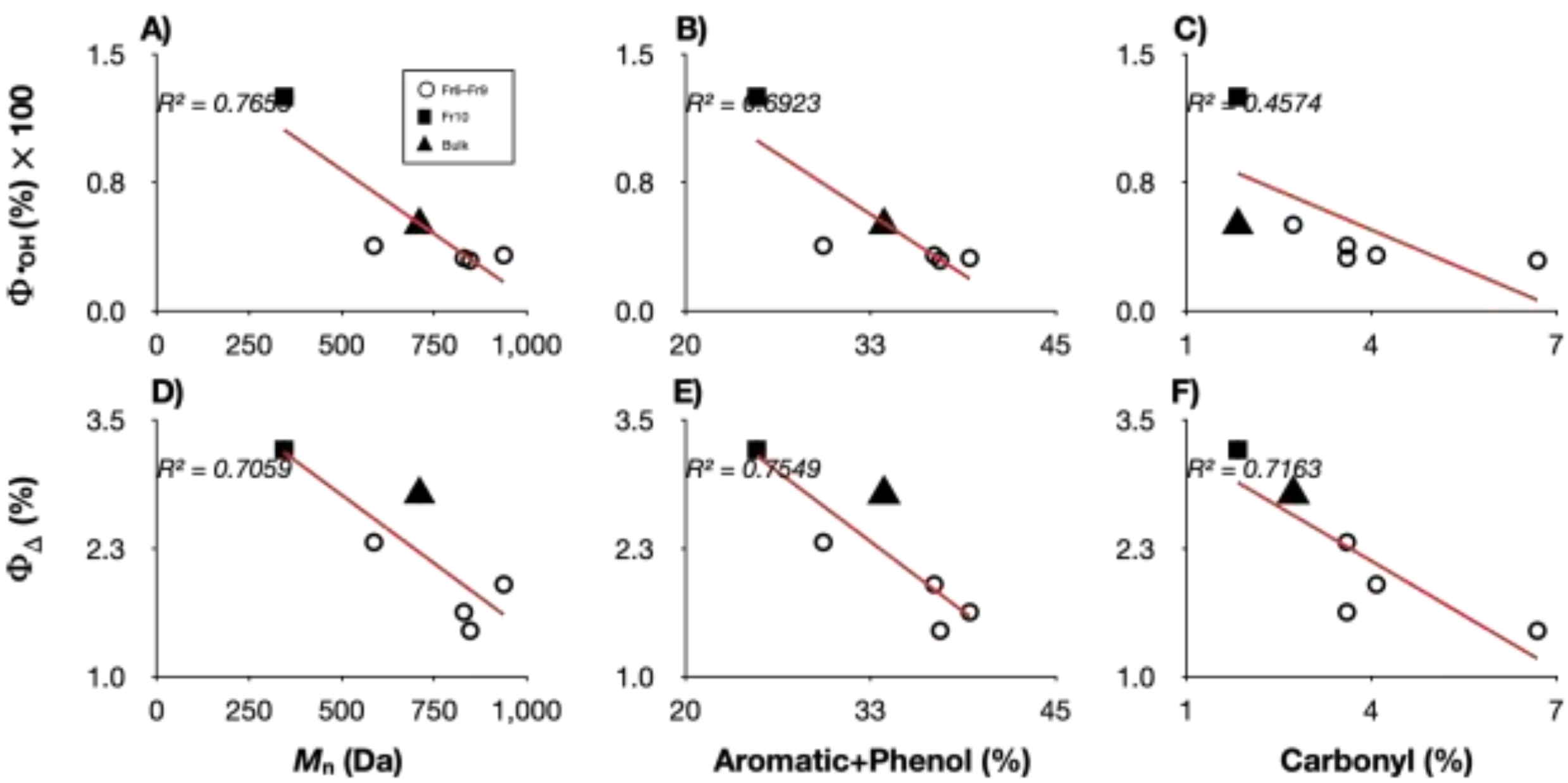

Figure S5. Scatter plots of hydroxyl radical and singlet oxygen quantum yields versus aromaticity, M_n, and carbonyl content.

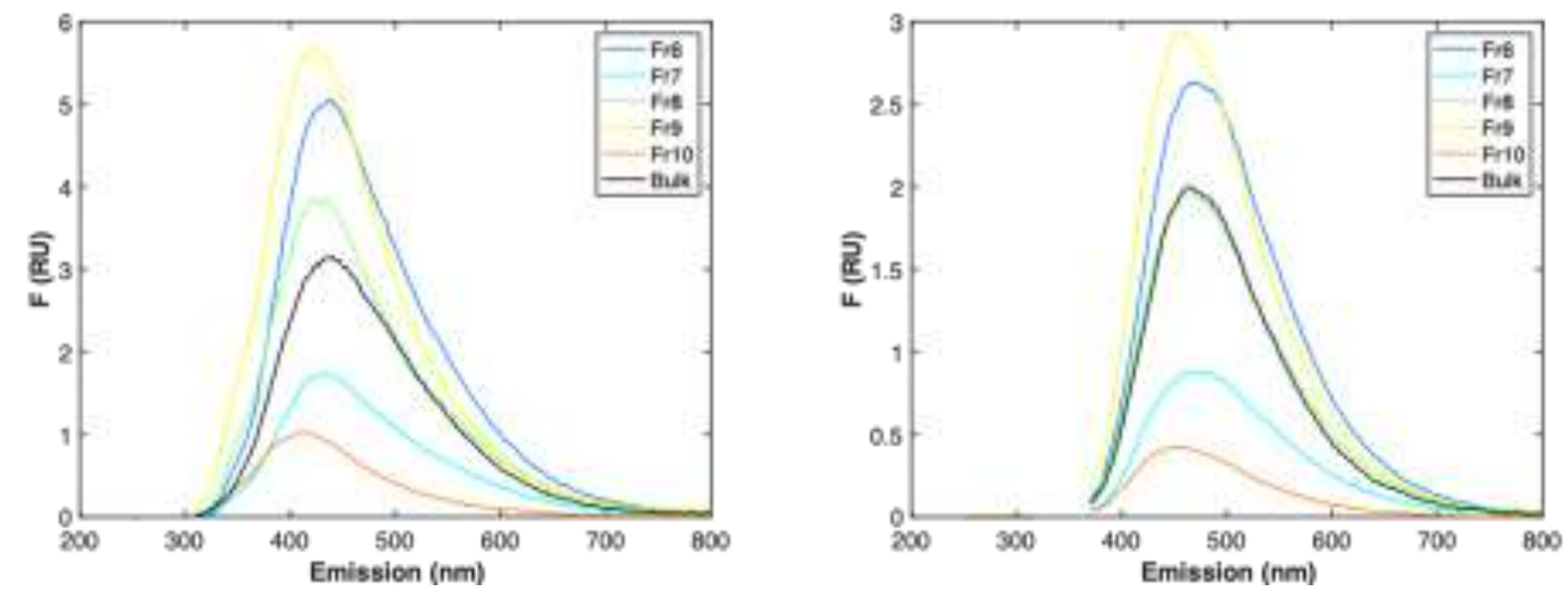

Figure S6. Emission spectra for Fr6–10 at (left) Ex=310 nm and (right) Ex=370 nm.

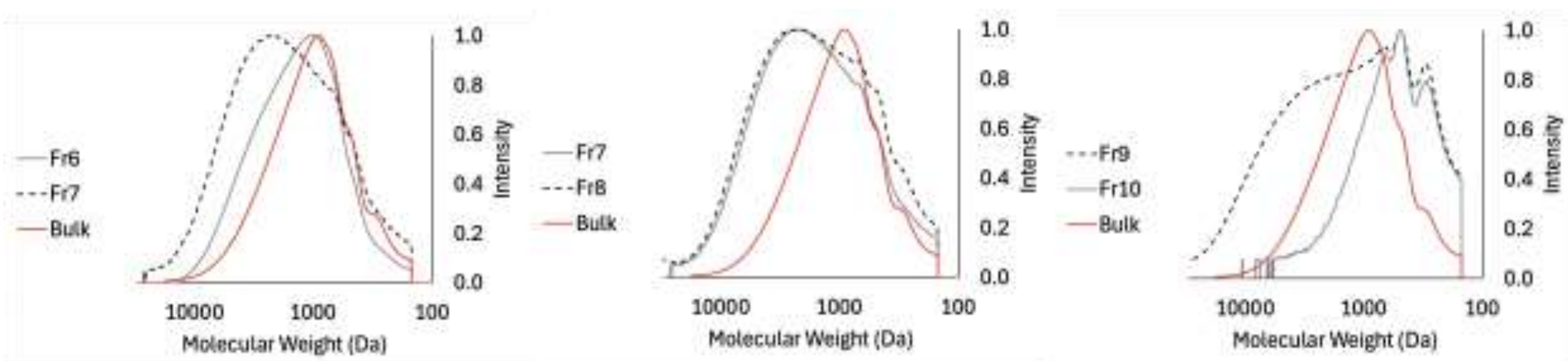

Figure S7. HP-SEC chromatograms for Fr6-Fr10 and bulk SRNOM.

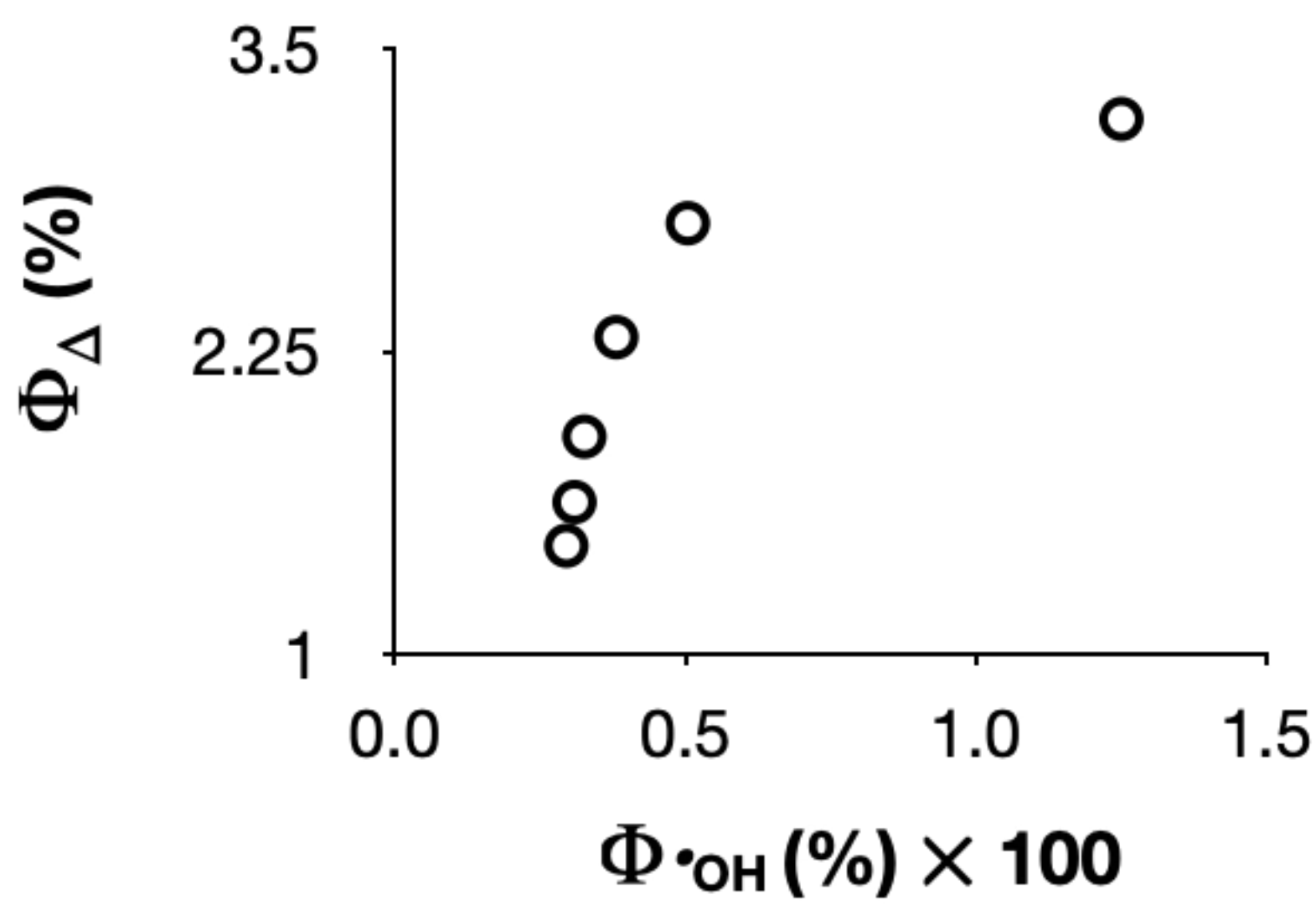

Figure S8. Relationship between singlet oxygen and hydroxyl radical quantum yields.

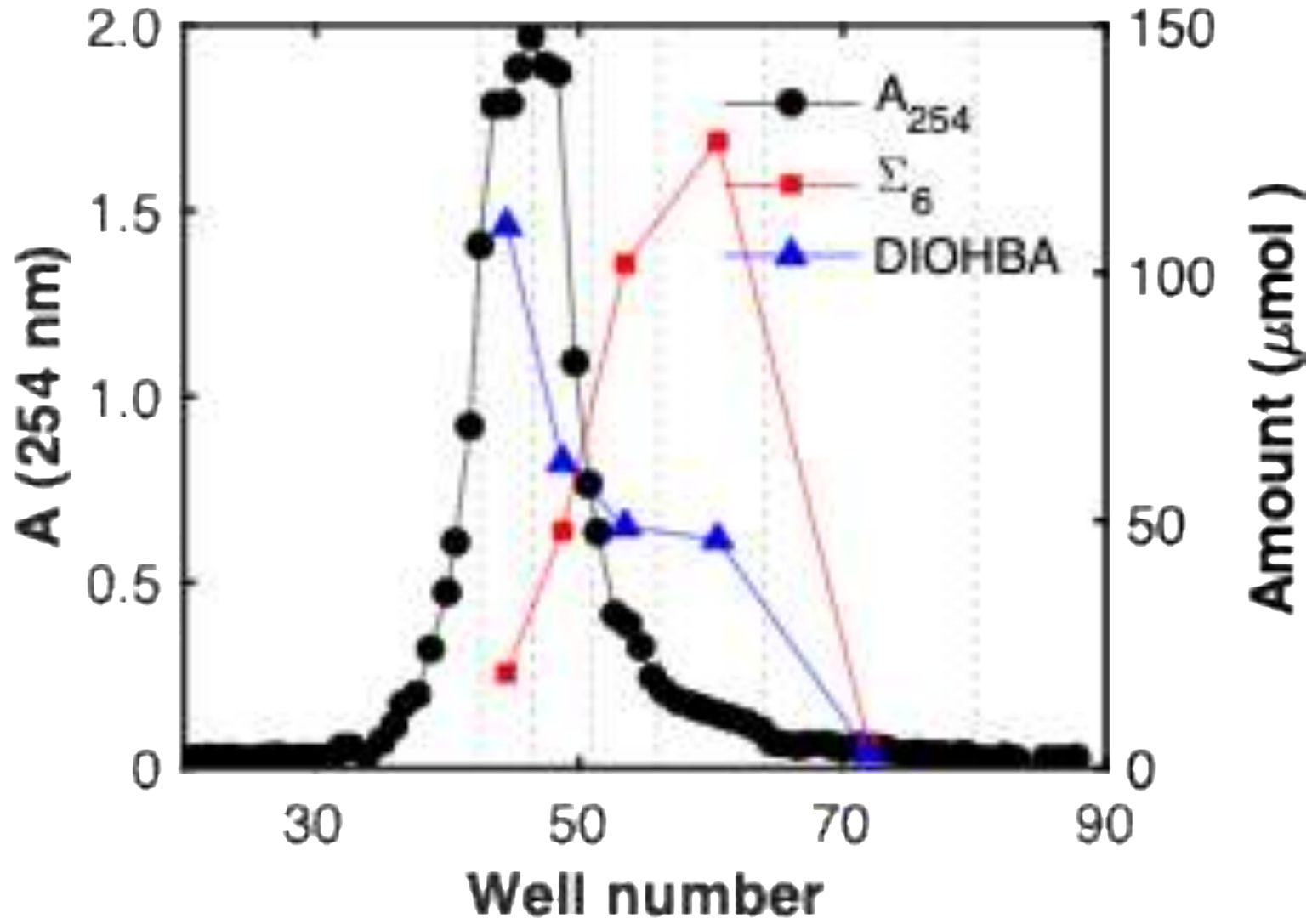

Figure S9. Electropherogram of SRNOM with absorbance at 254 nm detection. Right y-axis shows the amount in μmol of DIOHBA (3,5-dihydroxybenzoic acid) and Σ_6.

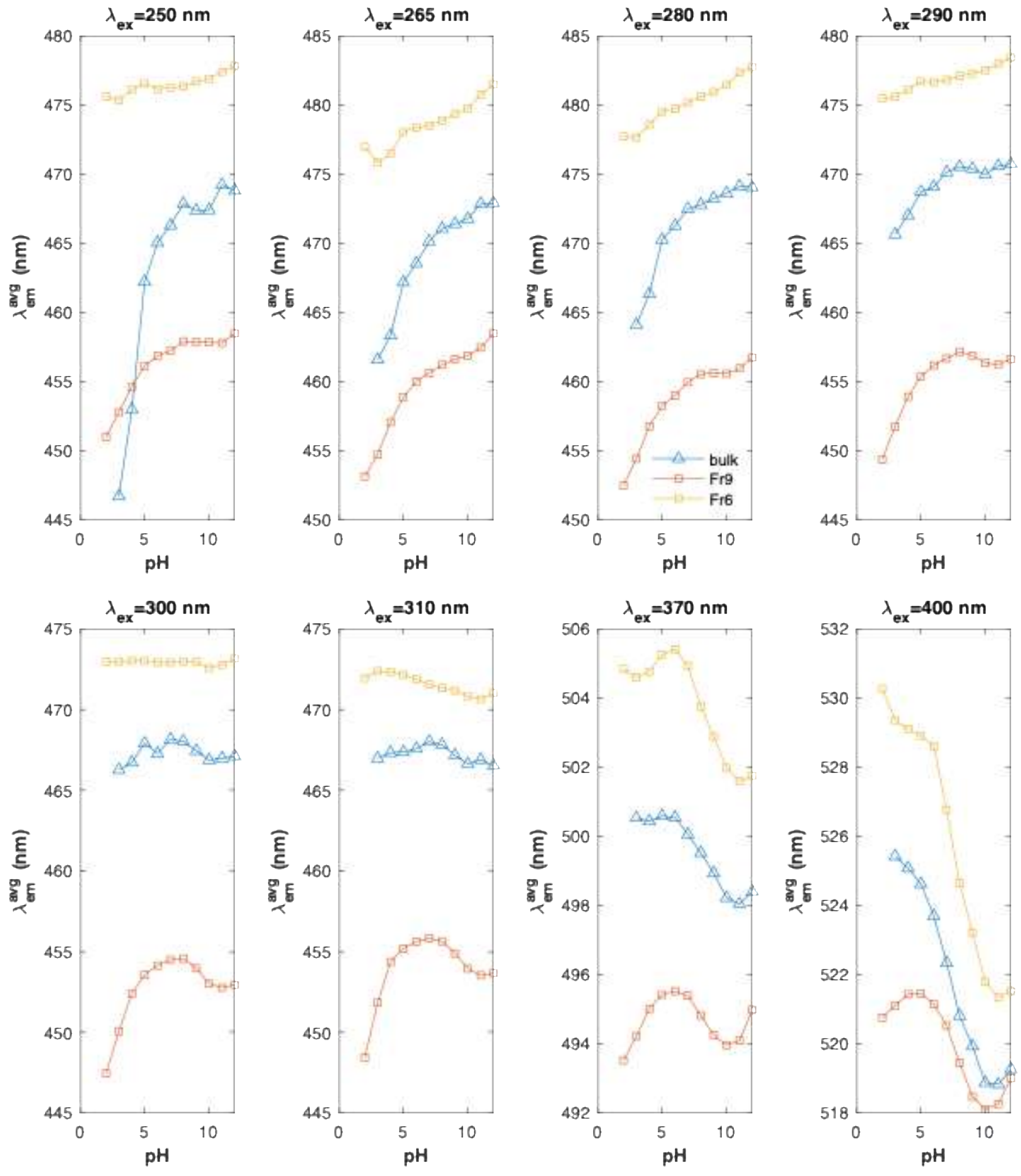

Figure S10. Intensity weighted emission as a function of pH for bulk SRNOM, Fr6 and Fr9. λ_{em}^{avg} was calculated as $\Sigma_{\lambda_{em}} F_{\lambda_{em}} \lambda_{em} / \Sigma_{\lambda_{em}} F_{\lambda_{em}}$ where $F_{\lambda_{em}}$ is the fluorescence intensity at emission wavelength λ_{em}.

(1) Gáspár, A. Unravelling the Interactions of Boron with Natural Organic Matter (NOM) on a Molecular Level, Technical University of Munich, 2008.

(2) Kaiser, K.; Benner, R. Hydrolysis-induced Racemization of Amino Acids. *Limnol. Oceanogr.: Methods* **2005**, *3* (8), 318–325. https://doi.org/10.4319/lom.2005.3.318.

(3) Kaiser, K.; Benner, R. Characterization of Lignin by Gas Chromatography and Mass Spectrometry Using a Simplified CuO Oxidation Method. *Anal. Chem.* **2012**, *84* (1), 459–464. https://doi.org/10.1021/ac202004r.

(4) McKay, G.; Korak, J. A.; Erickson, P. R.; Latch, D. E.; McNeill, K.; Rosario-Ortiz, F. L. The Case Against Charge Transfer Interactions in Dissolved Organic Matter Photophysics. *Environ. Sci. Technol.* **2018**, *52* (2), 406–414. https://doi.org/10.1021/acs.est.7b03589.

(5) Kothawala, D. N.; Murphy, K. R.; Stedmon, C. A.; Weyhenmeyer, G. A.; Tranvik, L. J. Inner Filter Correction of Dissolved Organic Matter Fluorescence. *Limnology and Oceanography: Methods* **2013**, *11* (12), 616–630. https://doi.org/10.4319/lom.2013.11.616.

(6) Korak, J. A.; McKay, G. Critical Review of Fluorescence and Absorbance Measurements as Surrogates for the Molecular Weight and Aromaticity of Dissolved Organic Matter. *Environ. Sci.: Process. Impacts* **2024**, *26* (10), 1663–1702. https://doi.org/10.1039/d4em00183d.

(7) Korak, J. A.; McKay, G. Meta-Analysis of Optical Surrogates for the Characterization of Dissolved Organic Matter. *Environ. Sci. Technol.* **2024**, *58* (17), 7380–7392. https://doi.org/10.1021/acs.est.3c10627.

(8) Schendorf, T. M.; Vecchio, R. D.; Bianca, M.; Blough, N. V. Combined Effects of pH and Borohydride Reduction on Optical Properties of Humic Substances (HS): A Comparison of Optical Models. *Environ. Sci. Technol.* **2019**, *53* (11), 6310–6319. https://doi.org/10.1021/acs.est.9b01516.

(9) Allen, A.; Cheng, K.; McKay, G. Evaluating the pH-Dependence of DOM Absorbance, Fluorescence, and Photochemical Production of Singlet Oxygen. *Environ. Sci.: Process. Impacts* **2023**, *25* (12), 1974–1985. https://doi.org/10.1039/d3em00316g.

(10) Li, H.; McKay, G. Fluorescence Quenching of Humic Substances and Natural Organic Matter by Nitroxide Free Radicals. *Environ Sci Technol* **2023**, *57* (1), 719–729. https://doi.org/10.1021/acs.est.2c02220.

(11) Green, S. A.; Morel, F. M. M.; Blough, N. V. Investigation of the Electrostatic Properties of Humic Substances by Fluorescence Quenching. *Environ. Sci. Technol.* **1992**, *26* (2), 294–302. https://doi.org/10.1021/es00026a008.

(12) Lakowicz, J. R. *Principles of Fluorescence Spectroscopy*, Third.; Springer, New York; Springer, New York, 1999.

(13) McAdams, B. C.; Aiken, G. R.; McKnight, D. M.; Arnold, W. A.; Chin, Y.-P. High Pressure Size Exclusion Chromatography (HPSEC) Determination of Dissolved Organic Matter Molecular Weight Revisited: Accounting for Changes in Stationary Phases, Analytical Standards, and Isolation Methods. *Environ. Sci. Technol.* **2018**, *52* (2), 722–730. https://doi.org/10.1021/acs.est.7b04401.

(14) Zhou, Q.; Cabaniss, S. E.; Maurice, P. A. Considerations in the Use of High-Pressure Size Exclusion Chromatography (HPSEC) for Determining Molecular Weights of Aquatic Humic Substances. *Water Res.* **2000**, *34* (14), 3505–3514. https://doi.org/10.1016/s0043-1354(00)00115-9.

(15) Laszakovits, J. R.; Berg, S. M.; Anderson, B. G.; O'Brien, J. E.; Wammer, K. H.; Sharpless, C. M. p-Nitroanisole/Pyridine and p-Nitroacetophenone/Pyridine Actinometers Revisited: Quantum Yield in Comparison to Ferrioxalate. *Environ. Sci. Technol. Lett.* **2017**, *4* (1), 11–14. https://doi.org/10.1021/acs.estlett.6b00422.

(16) Appiani, E.; Ossola, R.; Latch, D. E.; Erickson, P. R.; McNeill, K. Aqueous Singlet Oxygen Reaction Kinetics of Furfuryl Alcohol: Effect of Temperature, pH, and Salt Content. *Environ. Sci.: Process. Impacts* **2017**, *19* (4), 507–516. https://doi.org/10.1039/c6em00646a.
